# Seasonal variability of net sea-air CO_2_ fluxes in a coastal region of the northern Antarctic Peninsula

**DOI:** 10.1038/s41598-020-71814-0

**Published:** 2020-09-10

**Authors:** Thiago Monteiro, Rodrigo Kerr, Eunice da Costa Machado

**Affiliations:** 1 Programa de Pós-Graduação em Oceanologia, Instituto de Oceanografia, Universidade Federal do Rio Grande (FURG), Av. Itália km 8, Rio Grande, RS 96203-900 Brazil; 2 Laboratório de Estudos dos Oceanos e Clima, Instituto de Oceanografia, FURG, Rio Grande, RS Brazil; 3 Brazilian Ocean Acidification Network (BrOA), Rio Grande, RS Brazil; 4 Laboratório de Hidroquímica, Instituto de Oceanografia, FURG, Rio Grande, RS Brazil

**Keywords:** Biogeochemistry, Carbon cycle, Climate sciences, Biogeochemistry, Ocean sciences, Marine chemistry, Biogeochemistry

## Abstract

We show an annual overview of the sea-air CO_2_ exchanges and primary drivers in the Gerlache Strait, a hotspot for climate change that is ecologically important in the northern Antarctic Peninsula. In autumn and winter, episodic upwelling events increase the remineralized carbon in the sea surface, leading the region to act as a moderate or strong CO_2_ source to the atmosphere of up to 40 mmol m^–2^ day^–1^. During summer and late spring, photosynthesis decreases the CO_2_ partial pressure in the surface seawater, enhancing ocean CO_2_ uptake, which reaches values higher than − 40 mmol m^–2^ day^–1^. Thus, autumn/winter CO_2_ outgassing is nearly balanced by an only 4-month period of intense ocean CO_2_ ingassing during summer/spring. Hence, the estimated annual net sea-air CO_2_ flux from 2002 to 2017 was 1.24 ± 4.33 mmol m^–2^ day^–1^, opposing the common CO_2_ sink behaviour observed in other coastal regions around Antarctica. The main drivers of changes in the surface CO_2_ system in this region were total dissolved inorganic carbon and total alkalinity, revealing dominant influences of both physical and biological processes. These findings demonstrate the importance of Antarctica coastal zones as summer carbon sinks and emphasize the need to better understand local/regional seasonal sensitivity to the net CO_2_ flux effect on the Southern Ocean carbon cycle, especially considering the impacts caused by climate change.

## Introduction

The investigation of Antarctic coastal regions has long been neglected because they are difficult to access^1–4^, especially during periods other than the austral summer^5–8^. This occurs because of most of the year, i.e., from April to November, these regions are almost completely or completely covered by sea ice^9,10^. Such conditions lead to a biased representation of sampling in autumn and winter, which are likely critical periods for changes in seawater carbonate chemistry and net sea-air CO_2_ flux (FCO_2_). In fact, several studies have been conducted during the austral summer to better understand the FCO_2_^11–16^ and carbonate system parameter variability^17–22^ in the remote Southern Ocean. It is widely known that the Antarctic coasts behave as a strong CO_2_ sink during the summer^15,23^, which has intensified during recent years^14,15^. Actually, the intensity of this behaviour is marked by high interannual variability, since the summer CO_2_ fluxes in the Gerlache Strait, for example, oscillate between periods of strong CO_2_ sink (i.e., < − 12 mmol m^–2^ day^–1^) and sea-air near-equilibrium conditions at inter-annual scales^15^. However, even when Antarctic coastal regions do not behave as a strong CO_2_ sink, they take up CO_2_ in the summer^15^, although eventual episodes of CO_2_ outgassing can occur^20^.

Although some studies have provided important information on the seasonality of the FCO_2_^7,8,19^, they are restricted to a few specific years or localized regions, which may bias the modelled long-term trends of these regions. Hence, understanding the annual budget of sea-air CO_2_ exchanges remains a challenge^4,24^. This is particularly true for the Gerlache Strait and likely other major embayments around the Antarctic coasts, since it remains unclear whether this CO_2_ sink behaviour persists throughout the year or is balanced in other seasons. Moreover, little is known about the main drivers of FCO_2_ seasonality and their consequences for the sea surface carbonate system. Therefore, here, we present an annual overview of the FCO_2_ and the carbonate system properties in the Gerlache Strait, an ecologically and climatically important area of the northern Antarctic Peninsula (NAP). Furthermore, we demonstrate that this region acted as an annual net CO_2_ source to the atmosphere from 2002 to 2017, contrasting with previous findings for the western Antarctic Peninsula environments^7,8,19^ and other regions around Antarctica^25–27^.

## Oceanographic features of the Gerlache Strait

**Figure 1:**
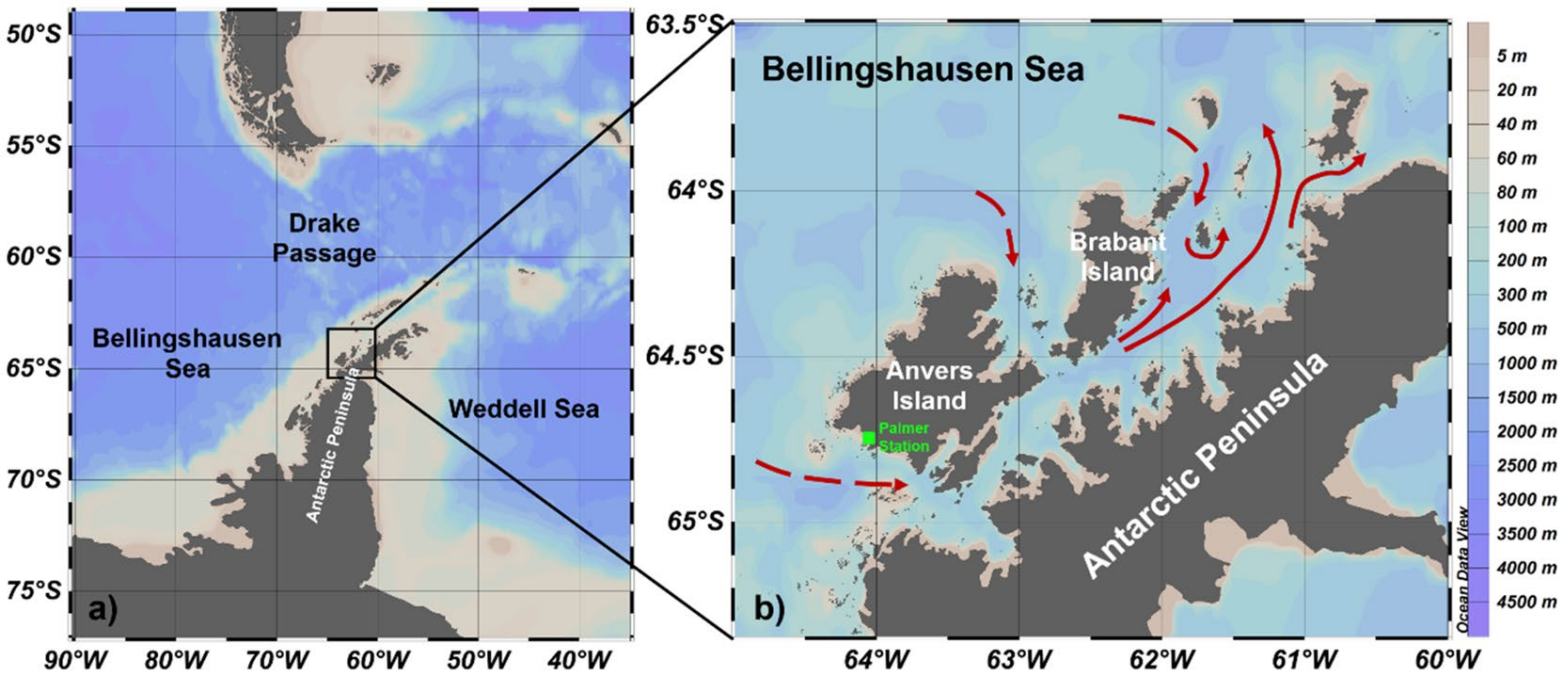
Location of the (**a**) western and northern Antarctic Peninsula and the (**b**) Gerlache Strait, with a simplified surface circulation pattern (red arrows) that is strongly influenced by the Bellingshausen Sea. The surface circulation in (**b**) was based on Savidge and Amft^98^. The dashed red arrows represent the modified Circumpolar Deep Water intrusions into the strait, which were identified by Smith et al.^42^, Prézelin et al^.^^36^ and García et al.^43^. The green square depicts the U.S. Palmer Station location (64.8°S, 64.1°W), from which we extracted atmospheric data. The colour shading represents the bottom bathymetry. These maps were generated by using the software Ocean Data View (v. 5.3.0, https://odv.awi.de)^100^.

The Gerlache Strait is a coastal region along the NAP that is being impacted by climate change^24,28^ and is essential for the health of the Antarctic food web^29–31^. The strait is a shallow basin that lies between the NAP and the Palmer Archipelago and is connected to the Bellingshausen Sea (to the west) and the Bransfield Strait (to the north) (Fig. 1a,b). Although it covers a smaller area (~ 8000 km^2^) than other coastal regions around the NAP, the Gerlache Strait is a highly productive coastal zone. In the Gerlache Strait, records of chlorophyll *a* (used as an indicator of primary producer biomass) range from ~ 2.0 mg m^–3^^31^ to ~ 23 mg m^–3^^32^ under distinct austral summer conditions. These concentrations have the same or a greater magnitude than those observed in more extensive regions, such as the Bransfield Strait (4.4 ± 3.84 mg m^–3^)^32^ and the northwestern Weddell Sea (1.38 ± 2.01 mg m^–3^)^33^. In addition, the Gerlache Strait has experienced intense diatom blooms reaching > 45 mg m^–3^ of chlorophyll *a*^32^. Although higher, this value is consistent with that recorded in the vicinity of Palmer Station, in the southernmost part of the Gerlache Strait, where the maximum chlorophyll *a* recorded was ~ 30 mg m^–3^^34^.

The high biological productivity in this region, reflected at different trophic levels^35^, is mainly due to the complex interplay of its distinct water mass sources, sea ice dynamics, ocean circulation, nutrient-rich meltwater input and protection from severe weather conditions^36,37^. Additionally, the rapid effects of climate change^24,28,38^, a recent increase in glacial meltwater discharge^39^, and likely the advection of both organic and anthropogenic carbon around the NAP^21,40,41^ have influenced the coupled physical-biological processes changing the carbon biogeochemistry across the entire western Antarctic Peninsula shelf region^28,39^.

Moreover, the Gerlache Strait is affected by irregular intrusions of Circumpolar Deep Water (CDW; e.g., Refs.^36,42–44^) (Fig. 1b). CDW is a warm, salty, poorly oxygenated and carbon- and nutrient-rich water mass flowing eastward with the Antarctic Circumpolar Current at intermediate and deep levels around the continent^43,45,46^. CDW intrusions along the western shelf of the Antarctic Peninsula are often associated with upwelling, mainly caused by shallow bathymetry^47^ and predominant wind systems^36^. These intrusions are also affected by modes of climate variability that regulate the intensity of winds in the Southern Ocean, such as the El Niño Southern Oscillation (ENSO) and Southern Annular Mode (SAM)^45,48,49^. During the positive phases of the SAM, the westerly winds are intensified, and the frequency and intensity of episodic CDW intrusions increase^45^. Conversely, under extreme ENSO, winds are weakened and cooled^48^, probably reducing CDW intrusions on the western shelf of the Antarctic Peninsula. Under any of these conditions, the physical properties of CDW change when it is mixed with cooler and less saline surface waters, originating the modified CDW (mCDW) in the shelf and coastal domain.

At depths greater than 100 m, the Gerlache Strait is influenced by the mixing of water masses sourced from the Bellingshausen and Weddell seas. In addition to mCDW, the north of the strait is influenced by a modified variety of High Salinity Shelf Water (HSSW), which is cooler and more oxygenated than CDW^45,50^. HSSW is formed on the northwestern Weddell Sea continental shelf and is advected towards and along the Bransfield Strait by the Antarctic Coastal Current^50,51^. Signs of its presence at deep levels of the Gerlache Strait are an important aspect of the NAP because HSSW is younger than CDW, and the biogeochemical impact of mixing between the modified varieties of these waters is not yet completely understood^21,22,40^. However, a consequence of HSSW is the intrusion of anthropogenic carbon in deep levels of the strait^25,26^, which can intensify the ocean acidification process in the region.

## Results

### Hydrographic properties and the carbonate system

**Figure 2:**
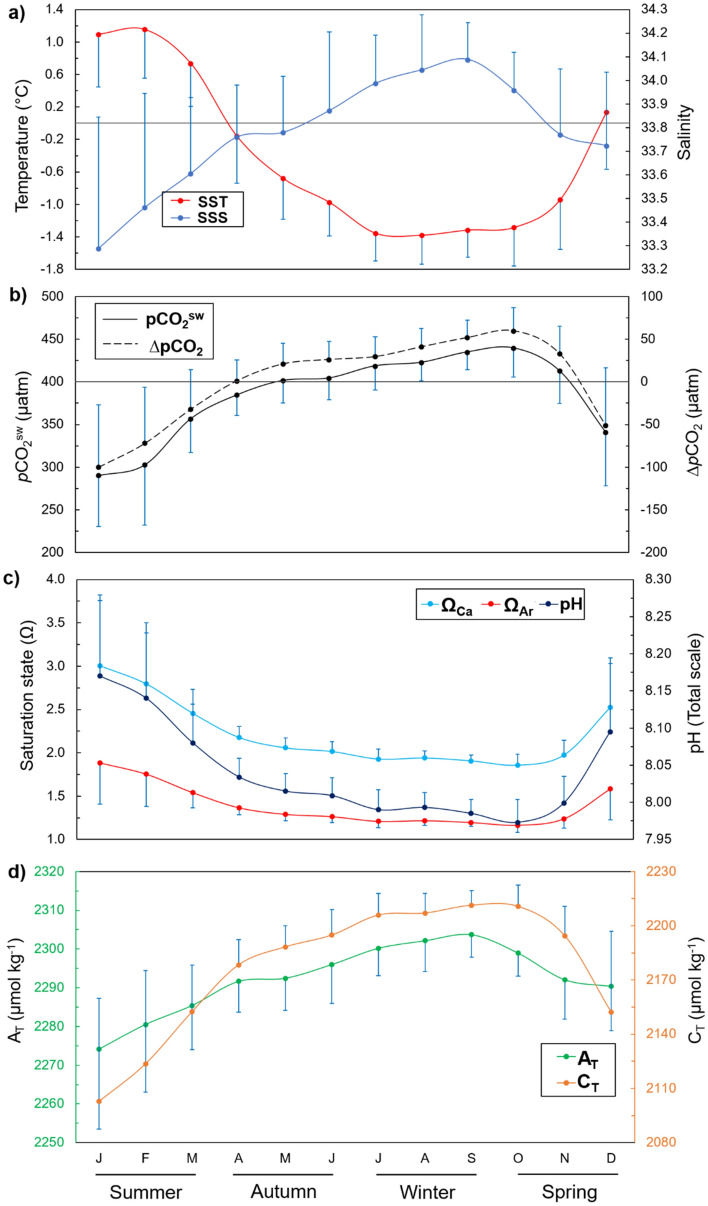
Detrended annual cycle of hydrographic and carbonate system properties on the surface of the Gerlache Strait. (**a**) Temperature and salinity, (**b**) CO_2_ partial pressure in the sea surface (*p*CO_2_^sw^) and the difference between *p*CO_2_^sw^ and atmospheric *p*CO_2_ (∆*p*CO_2_), (**c**) pH (total scale) and saturation states of calcite (Ω_Ca_) and aragonite (Ω_Ar_), and (**d**) total alkalinity (A_T_) and total dissolved inorganic carbon (C_T_). The blue bars are the standard deviations oriented up or down for visual clarity. The horizontal lines are the boundaries of 0 °C (**a**) and a ∆*p*CO_2_ equal to 0 (**b**).

Negative sea surface temperatures were recorded from April to November (Fig. 2a), and the lowest values in summer were observed in the northernmost part of the strait, where the highest salinities were recorded (Figure S4). The opposite temperature distribution pattern occurred during spring, when the lowest temperatures were recorded at the southern end of the strait. At the connection between the central basin of the Gerlache Strait and the Bellingshausen Sea (i.e., Schollaert Channel), higher temperatures were associated with lower salinity (Figure S4). On the other hand, the spatial distributions of temperature and salinity in autumn and winter were more homogeneous than those in summer and spring. The carbonate system properties also demonstrated distinct spatial distribution patterns among seasons (Figures S5–S8). The seasonal variabilities of total alkalinity (A_T_) and total dissolved inorganic carbon (C_T_) followed that of seawater CO_2_ partial pressure (*p*CO_2_^sw^) and were inverse to those of pH and the calcite and aragonite saturation states (Ω_Ca_ and Ω_Ar_, respectively) throughout the year. A_T_ was higher than C_T_ from December to March and was lower than C_T_ during the rest of the year (Fig. 2d). This seasonal pattern was also observed for CO_2_ saturation relative to the atmosphere; i.e., the difference (∆*p*CO_2_) between *p*CO_2_^sw^ and the CO_2_ partial pressure in the atmosphere (*p*CO_2_^atm^) was positive from April to November and negative from December to March (Fig. 2b). Minimum pH values (total scale) of 7.99 ± 0.02 were observed in winter, while in the other seasons, they were equal to or greater than 8.00 (Fig. 2c). Undersaturated carbonate calcium conditions (i.e., Ω less than 1) were not observed for either species during the seasonal cycle (Fig. 2c), although the lowest surface values of Ω_Ca_ and Ω_Ar_ were recorded in winter, on average.

**Figure 3:**
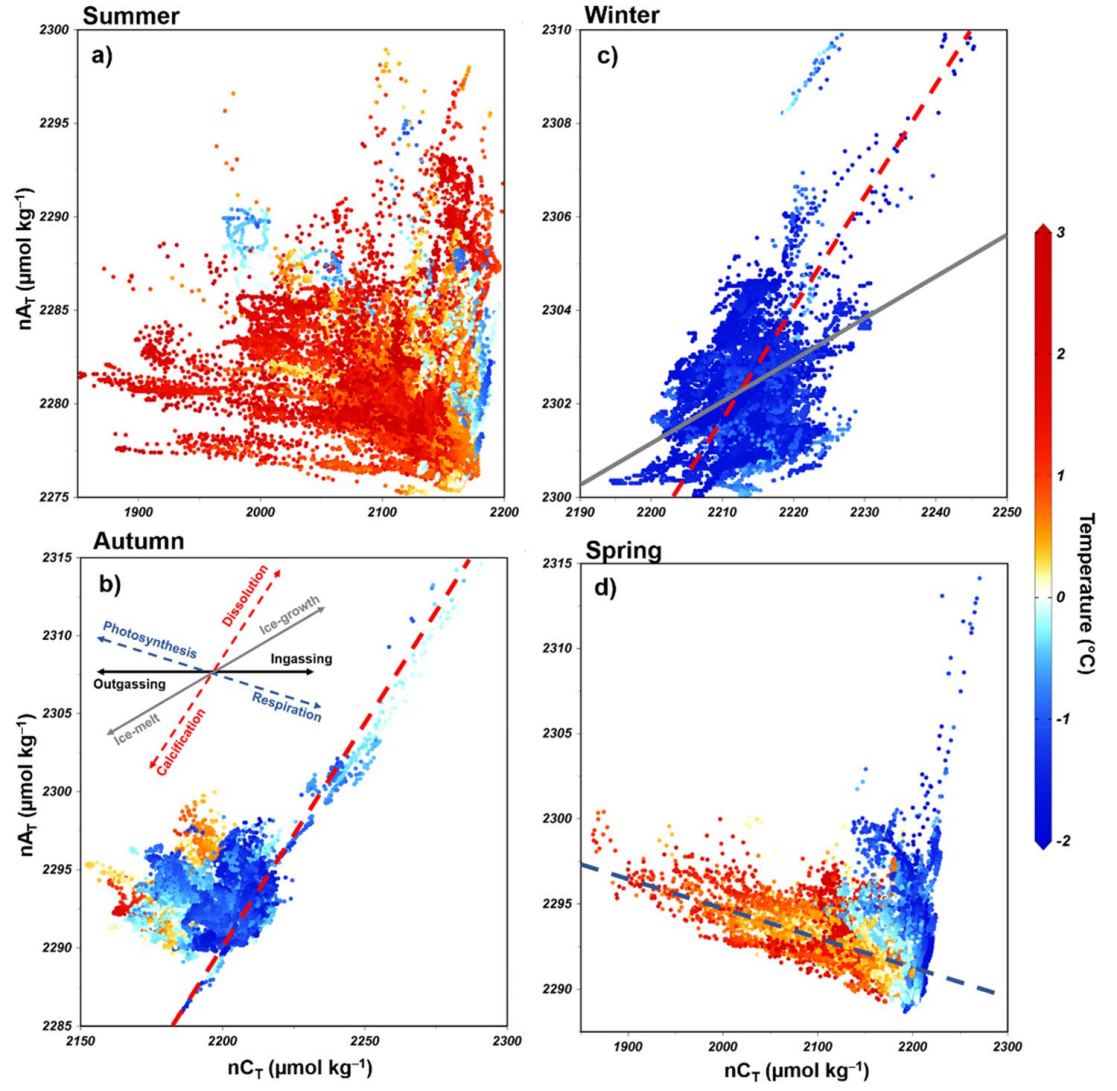
Salinity-normalized (average salinity for each season as in Figure S4) total alkalinity and total dissolved inorganic carbon (nA_T_ and nC_T_, respectively) dispersal diagram for the (**a**) summer, (**b**) autumn, (**c**) winter, and (**d**) spring. nA_T_ and nC_T_ were calculated for non-zero salinities following Friis et al.^99^. Arrows represent the nA_T_:nC_T_ ratio that characterizes the physical-biogeochemical processes that affect nA_T_ and nC_T_ (adapted from Zeebe^56^). The theoretical arrow representing the sea ice growth and melt processes was based on the threshold values for A_T_ and C_T_ described in Rysgaard et al.^64^. More details about the normalization of A_T_ and C_T_ as well as sea ice growth and melt processes are provided in the Supplementary Material. Note that the magnitudes of the axes are different among subplots.

In summer, virtually all processes exerted some influence on the surface CO_2_ system, as shown by the wide dispersion of the salinity-normalized A_T_ and C_T_ (nA_T_ and nC_T_, respectively; Fig. 3a). In general, carbonate dissolution seems to exert a greater influence in autumn and winter than in spring and summer, although sea ice growth also acts to control A_T_ and C_T_ in winter. Carbonate dissolution/calcification processes were observed to play a role in changing the A_T_ and C_T_ surface distributions in spring, although sea ice growth and melting processes are also expected to exert an influence, mainly during October and November, in association with low temperatures (Fig. 3d) and high *p*CO_2_^sw^. On the other hand, high temperatures (> 0 °C) in spring were associated with an increased influence of photosynthesis on the A_T_ and C_T_ (Fig. 3d).

### Drivers of *p*CO_2_^*sw*^ seasonal changes

**Figure 4:**
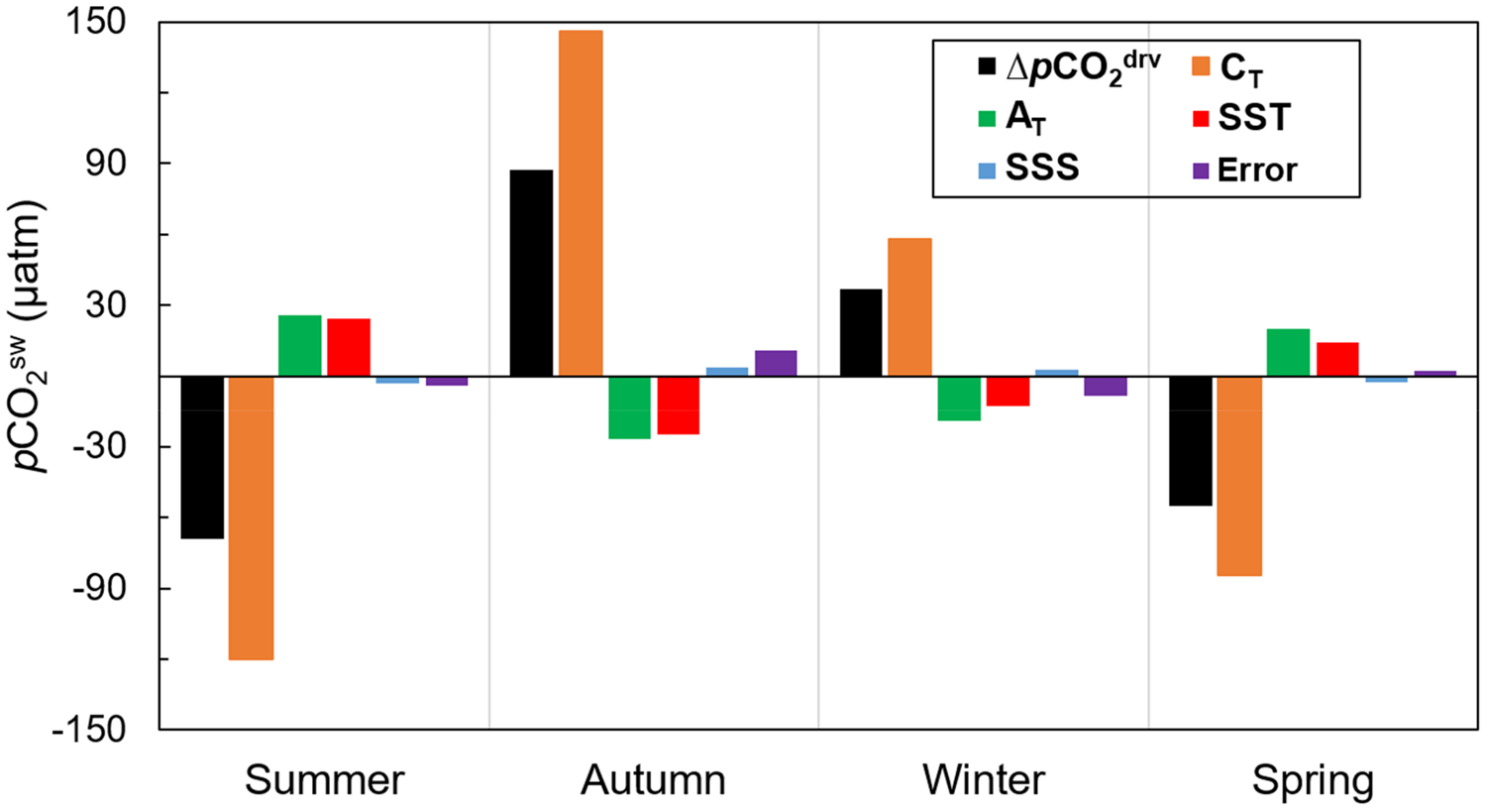
Effects of total alkalinity (A_T_), total dissolved inorganic carbon (C_T_), sea surface temperature (SST) and sea surface salinity (SSS) on seawater *p*CO2 (*p*CO_2_^sw^) for each season in the Gerlache Strait. The variation in each parameter is calculated as the difference between the values of each parameter and their respective averages in previous seasons. The unit of all drivers is the same as that for *p*CO_2_^sw^ (µatm), and their magnitudes represent their influence on *p*CO_2_^sw^ changes. Positive values indicate that an increase in the parameter led to an increase in *p*CO_2_^sw^; negative values indicate that a decrease in the parameter led to a decrease in *p*CO_2_^sw^. The only exception to this is A_T_ because an increase in A_T_ leads to a decrease in *p*CO_2_^sw^ and vice versa. The error bars (purple) show the difference between the sum of all drivers and the actual variation in *p*CO_2_^sw^ (∆*p*CO_2_^drv^), indicating the extent to which the decomposition of *p*CO_2_^sw^ into its drivers differs from Δ*p*CO_2_^drv^. More details are given in the methods section.

C_T_ had the dominant effect on changes in *p*CO_2_^sw^ throughout the year. A_T_ and temperature were secondary drivers of these changes, while salinity had a minor influence on surface *p*CO_2_^sw^ (Fig. 4). In summer and spring, there was a considerable decrease in *p*CO_2_^sw^, mainly due to the C_T_ drawdown. This decrease was compensated by the increasing effect on *p*CO_2_^sw^ of the reduction in A_T_ and the increase in temperature. In winter and autumn, the considerable increase in *p*CO_2_^sw^ was driven by the increase in C_T_ and partially compensated for by the increase in A_T_ and decrease in temperature.

### Net sea-air CO_2_ fluxes (FCO_2_)

**Figure 5:**
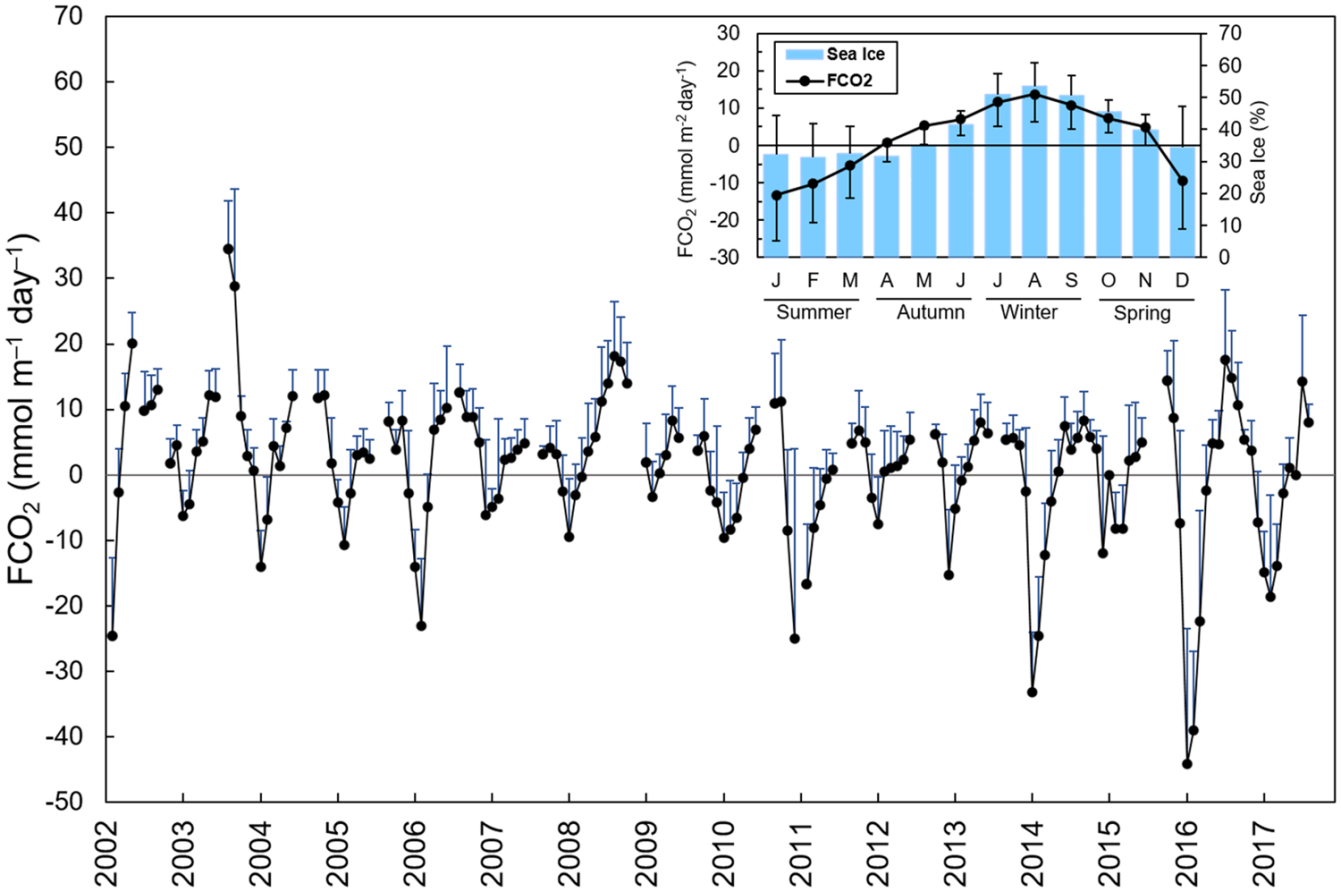
Monthly averages of net sea-air CO_2_ fluxes (FCO_2_) in the Gerlache Strait from January 2002 to December 2017 with an inset showing the variability throughout the year to characterize the seasonal cycle of FCO_2_ and the percentage of sea ice cover (filled blue bars). The gaps are from years when there was no winter sampling in the region. The blue bars oriented upwards are the standard deviations from the respective monthly averages, as are the black bars in the inset. Positive FCO_2_ values represent the outgassing of CO_2_ to the atmosphere, whereas negative FCO_2_ values represent CO_2_ uptake by the ocean.

FCO_2_ exhibited distinct seasonality throughout the year, with the region swinging from a strong CO_2_ sink (FCO_2_ < − 12 mmol m^–2^ day^–1^) in summer to a strong CO_2_ source (FCO_2_ > 12 mmol m^–2^ day^–1^) in winter (Fig. 5). During autumn and spring, the behaviour of the region oscillated between the major situations normally observed during winter and summer, resulting in a moderate FCO_2_. Despite this well-marked seasonality, the region was an annual weak CO_2_ source from 2002 to 2017, with an average estimated FCO_2_ of 1.24 ± 4.33 mmol m^–2^ day^–1^. Notably, with high spatial and temporal variability, this net near-equilibrium condition was achieved because the region switched from a moderate to strong CO_2_ ocean sink from December to March to a moderate to strong CO_2_ source to the atmosphere throughout the rest of the year (Fig. 5). Months with the most intense CO_2_ uptake levels (< − 12 mmol m^–2^ day^–1^) have occurred more frequently since 2011, with the peak in January and February of 2016. On the other hand, months with the maximum CO_2_ outgassing (> 12 mmol m^–2^ day^–1^) seem to have become less frequent since 2009 (Fig. 5).

**Figure 6:**
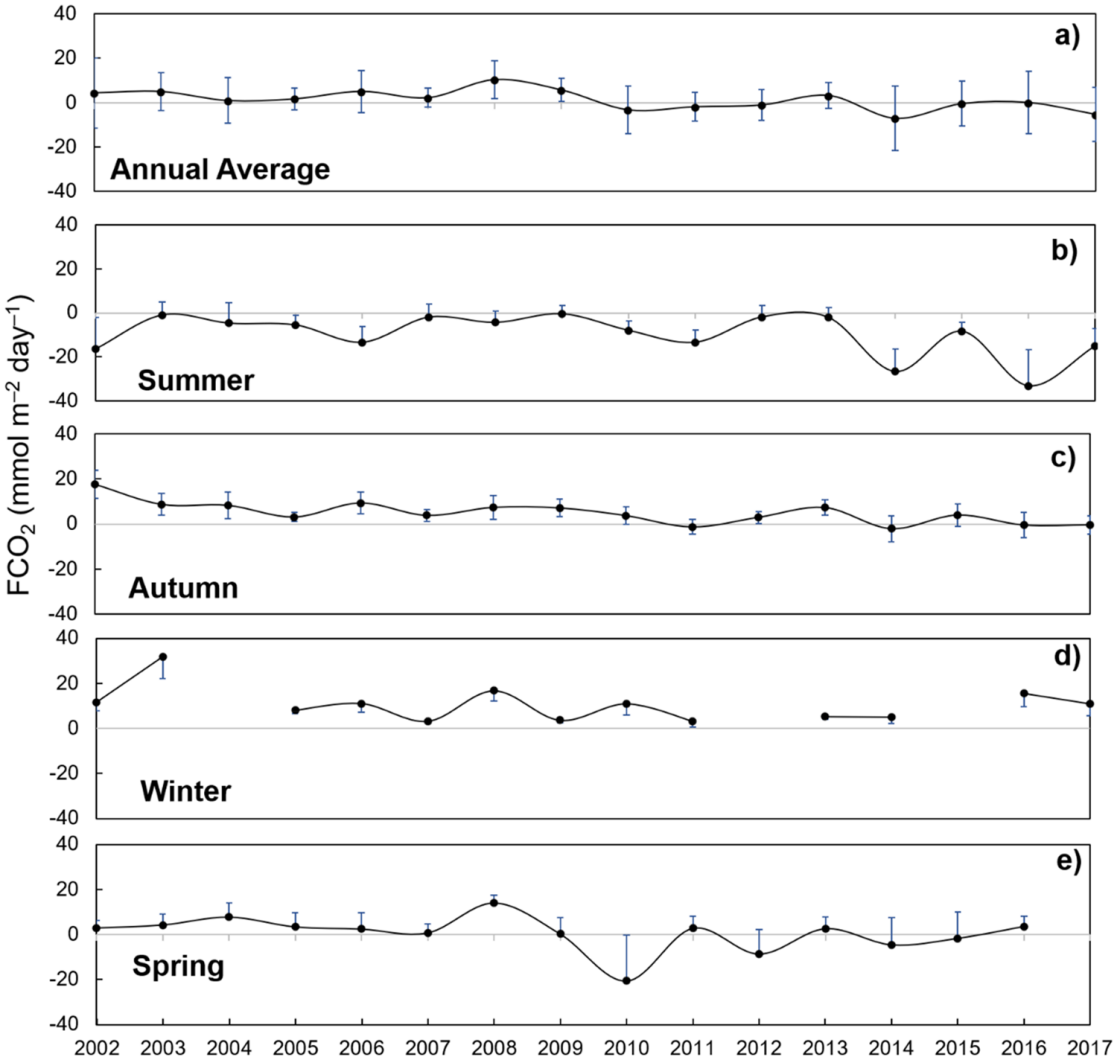
Time series of average (**a**) annual net sea-air CO_2_ flux (FCO_2_) during (**b**) summer, (**c**) autumn, (**d**) winter and (**e**) spring in the Gerlache Strait from 2002 to 2017. The gaps are from years when there was no winter sampling in the region. The blue bars oriented upwards are the standard deviations from the respective annual averages. Positive FCO_2_ values represent the outgassing of CO_2_ to the atmosphere, whereas negative values represent CO_2_ uptake by the ocean.

Considering all seasons between 2002 and 2017, high seasonal variability in FCO_2_ magnitude was identified (Fig. 6). However, the behaviour of the Gerlache Strait as a CO_2_ sink or source remained almost consistent within each season, as observed in summer (Fig. 6b) and winter (Fig. 6d). Only two particular exceptions occurred in the autumns of 2011 and 2014, when the region was a weak CO_2_ sink (Fig. 6c). Exceptions were also identified in spring, when the region behaved as a strong CO_2_ source in 2008 and a particularly strong CO_2_ sink in 2010 (Fig. 6e). Although the specific episodes in autumn did not appear to influence the average annual FCO_2_, the unusual spring FCO_2_ magnitudes coincided with increases in the average annual FCO_2_ in the respective years (Fig. 6a). The Gerlache Strait acted as an absolute annual CO_2_ source of 4.4 ± 2.8 mmol m^–2^ day^–1^ from 2002 to 2009 and has become predominantly a net annual CO_2_ sink of − 2.0 ± 3.0 mmol m^–2^ day^–1^ since 2010 (Fig. 6a).

**Figure 7:**
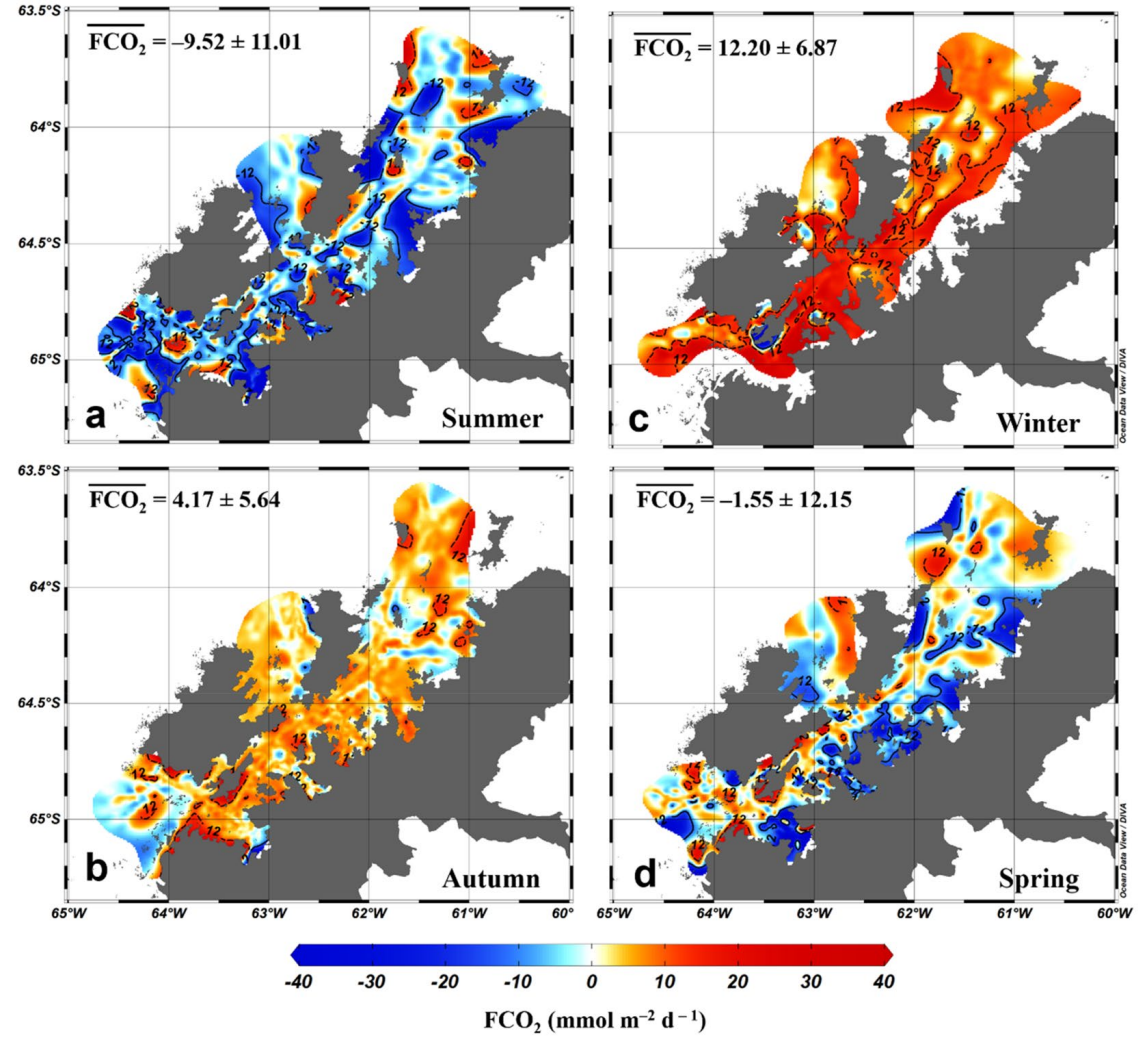
Surface distribution of the net sea-air CO_2_ flux (FCO_2_) in the Gerlache Strait from 2002 to 2017 in (**a**) summer, (**b**) autumn, (**c**) winter and (**d**) spring. Positive FCO_2_ values represent the outgassing of CO_2_ to the atmosphere, whereas negative FCO_2_ values represent CO_2_ uptake by the ocean. The numbers indicate the averages and standard deviations of FCO_2_ in each season. The black continuous and dashed isolines depict the FCO_2_ values of –12 and + 12 mmol m^–2^ d^–1^, respectively, for the strong CO_2_ sink and outgassing situations. These maps were generated by using the software Ocean Data View (v. 5.3.0, https://odv.awi.de)^100^.

A seasonal pattern in the spatial distribution of FCO_2_ along the Gerlache Strait was also identified. This pattern was characterized by a more homogeneous spatial distribution in autumn and winter (Fig. 7b,c) than in summer and spring (Fig. 7a,d). Moreover, the northernmost part of the strait, north of 64°S, had a higher annual FCO_2_ (3 ± 8 mmol m^–2^ day^–1^) than the southernmost part of the strait, south of 65°S. In the southernmost part, there was an annual CO_2_ uptake of − 7 ± 16 mmol m^–2^ day^–1^.

## Discussion

### Seasonal changes in sea-air CO_2_ fluxes

**Figure 8:**
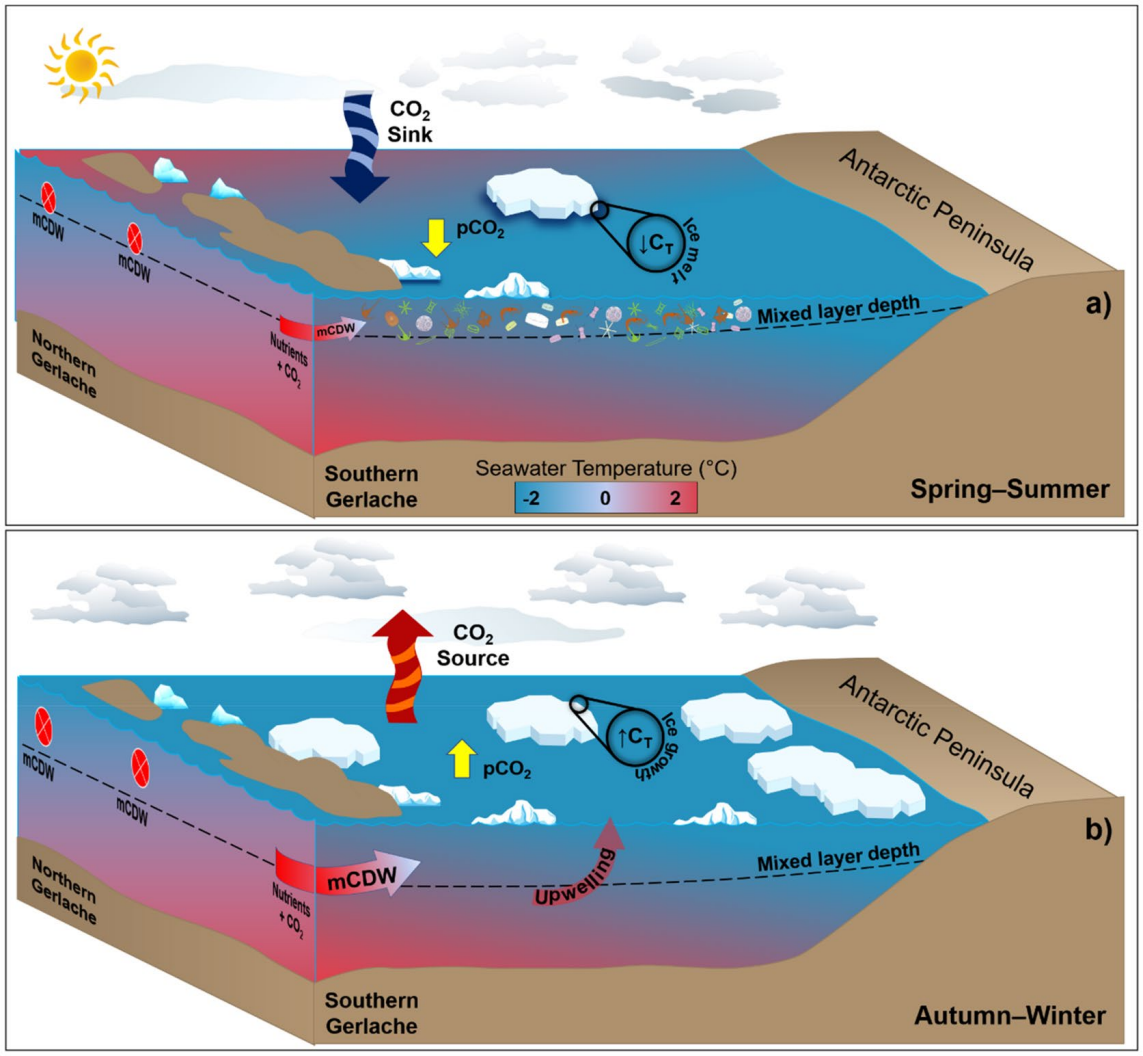
Distinct processes driving surface CO_2_ partial pressure (*p*CO_2_) and seasonal sea-air CO_2_ fluxes in a coastal region of the northern Antarctic Peninsula (NAP). From (**a**) December to March, sea ice melting provides a shallow mixed layer that leads to phytoplankton growth. This spring–summer scenario coupled with less intense modified Circumpolar Deep Water (mCDW) intrusions into the NAP and a decrease in total dissolved inorganic carbon (C_T_) from meltwater causes *p*CO_2_ drawdown. Therefore, in these months, the region behaves as a strong sink of atmospheric CO_2_. Conversely, from (**b**) April to November, under sea ice cover conditions, more intense mCDW intrusions coupled with a deeper mixed layer lead to intensified vertical mixing, resulting in the upwelling of CO_2_-rich waters. Such processes, in association with the rejection of C_T_ through brine release during sea ice growth, lead to a significant increase in surface *p*CO_2_. Then, the region becomes a moderate to strong CO_2_ source to the atmosphere during the autumn–winter. The theoretical depth of the shallowest spring–summer mixed layer is approximately 50 m, reaching approximately 150 m in the autumn–winter^61^. Drawn by Thiago Monteiro. Symbols courtesy of the Integration and Application Network, University of Maryland Center for Envrionmental Science (ian.umces.edu/symbols/).

In late spring and summer, the Gerlache Strait is a CO_2_ sink, with rates ranging from − 13 ± 12 mmol m^–2^ day^–1^ in January to − 5 ± 9 mmol m^–2^ day^–1^ in March (Fig. 5). This strong CO_2_ uptake is driven by an increase in biological activity coupled with meltwater input (Fig. 8a)^14,15,20,52–54^ from December until late summer (Fig. 5), when sea ice formation becomes gradually more intense^9,10^. This is revealed by the substantial C_T_ drawdown (Fig. 4), which characterizes the influence of photosynthesis on the surface water^3,55,56^, associated with a slight decrease in A_T_ as a result of further respiration (Fig. 3b). Phytoplankton growth is favoured by the increased stability of the nutrient-rich shallower mixed layer in summer and late spring (Fig. 8a), mainly due to meltwater input^14, 46,53,54,57^. This is more evident in the southernmost part of the strait, where intrusions of warmer mCDW would likely lead to sea ice melting^36^ and the higher percentage of meteoric water (Figure S9) than in the northernmost region, which is comparatively ice-free (Figure S9). Hence, this could potentially account for the greater CO_2_ uptake in the southern region than in the northern region (Fig. 7). Nevertheless, the spatial variability of the carbonate system parameters is clearly greater in spring and summer than in autumn and winter. Therefore, it is likely that other oceanographic processes simultaneously have roles in changing the surface nA_T_ and nC_T_.

In fact, during early spring, the carbonate dissolution/precipitation and sea ice growth/melt associated with low temperatures (Fig. 3d) seem to influence the carbonate system due to the increase in C_T_ that is rejected through the sea ice brine. However, the impact of each of these processes, and even the presence of other involved processes, is not yet well understood. The dominant processes in spring (i.e., carbonate dissolution/precipitation or photosynthesis/respiration), as well as during other seasons, can also exhibit interannual variability. For example, during summer, there is variability in CO_2_ uptake oscillating between 2 and 4 years, by which FCO_2_ in the region alternates between strong CO_2_ sink and near-equilibrium conditions^15^. This variability is associated with both intense biological activity and the intrusion of local upwelled CO_2_-rich waters (e.g., mCDW). In addition, it is linked to the influence of modes of climate variability, such as ENSO, which decreases the wind intensity, leading to favourable conditions for phytoplankton blooms^14^. This explains why the most intense CO_2_ uptake was recorded in 2016 (Fig. 5), as this was the year with the most extreme ENSO since 1998^58^, which was associated with biogeochemical changes along the water column^41^. Therefore, the same mechanism underlying the shift in the dominant physical processes may occur in other seasons of the year. This would likely explain why the region was an exceptionally strong CO_2_ source in spring 2008 but a strong CO_2_ sink in spring 2010 (Fig. 6e).

In autumn, the region becomes a moderate CO_2_ source to the atmosphere, with the maximum magnitude in August (14 ± 7 mmol m^–2^ day^–1^). Such behaviour is due to a significant increase in C_T_, which leads to an increase in *p*CO_2_^sw^. This is further partially offset by the effect that the increase in A_T_ has on *p*CO_2_^sw^ (Fig. 4), implicating the upwelling process as a likely cause. In fact, more intense short-term irregular intrusions of mCDW^44,59,60^ coupled to the deeper mixed layer, which lead to intensified vertical mixing in the winter^61^, are likely to carry CO_2_-rich waters to the surface layer of the strait (Fig. 8b). Indeed, this has been the process most observed in other Southern Ocean coastal regions^8,11,24^. On the western Antarctic Peninsula shelf, for example, there is no evidence of inorganic macronutrient regeneration in late summer, revealing that the increase in C_T_ must be more associated with upwelling and/or advection processes^18^. Although these mCDW intrusions can occur throughout the year and through virtually all connections of the Gerlache Strait^36,42,43^, they are expected to be more intense in winter^61^ and at the southern end of the strait^62^. In addition, the rejection of C_T_ through sea ice brine^63,64^ is an important process (Fig. 8b). Despite occurring more intensely in winter than in other seasons, this process should also contribute to CO_2_ release in autumn, as it was also dominant in controlling A_T_ and C_T_ (Fig. 3c). The increase in C_T_ due to ice growth, first shown in a laboratory experiment^63^, occurs in both Arctic and Antarctic regions, where there is an intense sea ice dynamic^64^. Hence, the increase in C_T_ leads to high *p*CO_2_^sw^ values but is also related to decreases in Ω_Ca_ and Ω_Ar_^65^. Thus, these conditions contribute to maintaining a relatively low pH (≤ 8.00) until mid-spring, when sea ice begins to melt and both C_T_ and *p*CO_2_^sw^ decrease towards the summer season.

Although the spatial distribution of FCO_2_ is more homogeneous in autumn and winter than in other seasons (Fig. 7), there is intense interannual variability in these fluxes (Fig. 6). It is not yet clear what drives this variability, but it has been linked to sea ice cover variability in other Antarctic regions^8,13,66^. This link makes sense due to the good correlation (*r*^2^ = 0.73; *p* = 0.0006; n = 12) of the FCO_2_ seasonal cycle with the sea ice cover seasonality in the Gerlache Strait, mainly in the months when it acts as a CO_2_ source (*r*^2^ = 0.93; *p* = 0.0136; n = 7) (Figure S10). Despite the strong CO_2_ outgassing during these periods, sea ice cover constrains sea-air CO_2_ exchanges^8,26^, leading to the conclusion that this CO_2_ outgassing could be even more intense under sea ice-free conditions, as observed in the Arctic Ocean^67^. Hence, the FCO_2_ dynamics in sea ice-covered periods may be more sensitive than previously thought.

### Seasonality of the carbonate system and acidification process

The carbonate system parameters on the surface of the strait follow seasonal FCO_2_ dynamics, that is, sea ice dynamics. The lower pH, Ω_Ca_ and Ω_Ar_ values in winter than in other seasons, although expected, reinforce the biogeochemical sensitivity of this season. The low temperatures and the brine released by sea ice growth lead to the dissolution of calcium carbonate and decreases in Ω_Ca_ and Ω_Ar_^19^. However, we did not find the calcium carbonate in the surface of the Gerlache Strait to be in a subsaturated state, even in winter when there was high *p*CO_2_^sw^; this was also the case in Ryder Bay^18,19^, a region located farther south on the western Antarctic Peninsula shelf, which is under dynamic conditions similar to those of the Gerlache Strait. In summer, carbonate mineral supersaturation is associated with regions where there is strong CO_2_ uptake, such as in the southernmost portion of the strait, where meteoric water input is most intense (Figure S9) and salinity is relatively low (Figures S4 and S7). This reveals that the intense *p*CO_2_^sw^ drawdown caused by biological activity outweighs the increase in *p*CO_2_^sw^ by the effect of carbonate precipitation^18^, and carbonate dissolution is minimized due to the biological uptake of C_T_. Nevertheless, the sensitivity of these parameters should be observed in more detail, as carbonate calcification and dissolution processes also seem to play an important role in controlling A_T_ and C_T_ (Fig. 3b,c). Furthermore, because we found minimum pH values in winter (7.92) lower than those at Ryder Bay in 1994 (8.11) and 2010 (8.00)^7^ as well as between 2011 and 2014 (7.95)^19^, these waters may be experiencing ocean acidification, although counterintuitive processes may be offsetting the effects in the studied region^22^. In fact, the waters of the Gerlache Strait have previously been reported to show signs of acidification in summer below the mixed layer^20,22^, with surface pH values lower than those found at Ryder Bay (8.21–8.48^18^).

The effects of intensified summer CO_2_ uptake on calcite and aragonite saturation in surface waters may emerge in the coming years. However, supersaturation of these carbonate species is associated with decreased *p*CO_2_^sw^ values in summer^15^. This reveals that these feedback effects need to be further investigated, especially considering the residence time of these waters in coastal regions. As strong summer CO_2_ sink periods are extended, an inverse effect of sea surface acidification may occur, as observed in the southernmost portion of the Gerlache Strait. Nevertheless, the acidification process should occur in the deep layers of these strong CO_2_ sink regions and in adjacent deep waters due to horizontal advection. Indeed, this will likely be the case because the residence time of surface waters in this region was estimated to be less than 7 days, while the residence time in adjacent larger basins ranges between 13 and 40 days^50^. Therefore, assuming a steady increase in both atmospheric CO_2_^68^ and temperature^69^, the Southern Ocean coastal regions may become intense hotspots of deep-ocean acidification, with some expected implications for organisms throughout the water column and the food web as a whole. For example, on the sea surface, there may be a restructuring of the food web due to a shift in the dominant groups of phytoplankton, such as from diatoms to smaller organisms [Refs.^24,53^ and references therein]. Such changes will potentially decrease the transfer of carbon, energy and nutrients through organisms such as diatoms to pelagic and benthic ecosystems, with complex feedbacks on ocean biogeochemistry and climate^24^. In this sense, these findings shed light on the importance of clarifying the real impacts of these changes throughout the water column. This is because, despite showing signs of acidification, most studies provide only snapshots, and coupled ocean–land–ice processes can mask the real ocean acidification state of Southern Ocean coastal regions.

### Annual budget of sea-air CO_2_ exchanges

We have identified the Gerlache Strait as a weak CO_2_ source from 2002 to 2017, with an annual budget of sea-air CO_2_ exchanges at near-equilibrium conditions. This contrasts with the expectations for other Antarctic coastal regions, which demonstrate annual CO_2_ sink behaviour^5,13,25^, such as in summer and spring^11,14,33^. The studied region acts as a moderate CO_2_ source in autumn and a strong CO_2_ source in winter. The CO_2_ outgassing that occurs during 8 months of the year (i.e., from April to November) is almost fully compensated for in only 4 months (i.e., from December to March), when the region acts as a moderate to strong CO_2_ sink. Although this behaviour is not considered typical for Antarctic coastal regions, the Gerlache Strait lies at approximately 64°S, where Takahashi et al.^70^ verified an approximately neutral annual sea-air CO_2_ flux. Nevertheless, here, we hypothesize that this scenario is more common to coastal regions of the Southern Ocean than previously thought because incipient signs of this behaviour have already been identified in other Antarctic coastal regions. For example, Bakker et al.^26^ found strong supersaturation of seawater CO_2_ relative to atmospheric CO_2_ in autumn and winter in the Weddell Sea but suggested that the region was an annual CO_2_ sink. These contrasting summer/winter behaviours, with an annual CO_2_ sink budget, also extend to other Southern Ocean coastal regions, such as the western Antarctic Peninsula^5,7,8,11^, the Ross Sea^13^, the Indian Antarctic sector^6,71^ and even the Antarctic Zone south of 62°S as a whole^72^. However, the relatively low monthly and interannual coverage in most of these studies may have biased the integrated FCO_2_ budget throughout the year. This is particularly true if we take into account recent estimates of FCO_2_ from long-term climatology for global coastal regions^4^. In this climatology, the NAP, as well as the Weddell Sea and much of the Atlantic and Indian sectors of the Southern Ocean, was a net CO_2_ source between 1998 and 2015. Despite this, the CO_2_ uptake by CO_2_ sink regions was so intense that the annual FCO_2_ budget for this period was approximately − 17 Tg C year^–1^^4^.

### Expected scenarios for the future of sea-air CO_2_ exchanges

The recent changes observed in the NAP, mainly related to the intensification of the westerly winds^49^, rising temperatures^73^ and the prolongation of ice-free water periods^74,75^, are expected to persist in the coming years^24,28^. In this sense, two future scenarios for net sea-air CO_2_ fluxes can be projected. First, with longer ice-free water periods, these coastal regions could release CO_2_ that would otherwise remain in the seawater isolated by sea ice, intensifying the annual CO_2_ source. This release may be enhanced by intensified mCDW intrusion into the western Antarctic Peninsula shelf that have been projected^24,45^, although little is known about its periodicity and variability. On the other hand, nutrient-rich mCDW intrusions coupled with the delayed sea ice cover period and rising temperatures should lead to prolonged phytoplankton growth^75^. Thus, strong CO_2_ sink periods should also extend beyond late summer. As CO_2_ uptake has intensified in the summer^14,15^ and proved to nearly counteract annual CO_2_ evasion, this region could become an annual CO_2_ sink in future years, particularly assuming that the Southern Ocean is becoming greener^75^. Actually, this second scenario seems likely to occur, as the magnitude and frequency of FCO_2_ in months when the region is a strong CO_2_ sink are increasing and in months when the region is a strong CO_2_ source have been less frequent (Fig. 5), leading to intensified annual CO_2_ uptake since 2010 (Fig. 6a).

These scenarios become more complex when we take into account the influence of the modes of climate variability. For example, the positive phase of SAM has been associated with more intense CO_2_ outgassing due to the deepening of the mixed layer^76^. Conversely, it was also associated with higher CO_2_ uptake due to the intensification of upwelling, which supplies iron and nutrients to the sea surface and hence increases phytoplankton growth^77^. This reveals the sensitivity of sea-air CO_2_ exchanges to these feedback mechanisms and the urgent need to broaden investigations for a coupled analysis of ocean-climate systems. Nevertheless, signs of intensifying summer CO_2_ sink behaviour^14,15^ suggest that the influence of SAM should be reversing the flux to encourage annual net CO_2_ uptake in Antarctic coastal regions.

## Methods

### Dataset and carbonate system properties

We used the data available from Surface Ocean CO_2_ Atlas version 6 (SOCATv6)^78^ to compile a temporal series spanning 2002 to 2017 (Figure S2) of the sea surface (up to a depth of 5 m) temperature (SST), salinity (SSS) and seawater CO_2_ partial pressure (*p*CO_2_^sw^) of the Gerlache Strait. Here, we evaluated the seasonal variability of the net sea-air CO_2_ flux (FCO_2_) and hydrographic and carbonate system parameters. Therefore, the seasons were defined as (1) summer: January to March; (2) autumn: April to June; (3) winter: July to September; and (4) spring: October to December. We analysed the months in which the data covered the majority of the Gerlache Strait in all seasons (Figure S3).

The *p*CO_2_^sw^ data extracted from SOCATv6 were directly measured using air–water equilibrators and an infrared analyser for CO_2_ quantification^78^. However, SOCATv6 provides surface *p*CO_2_^sw^ data with only corresponding SST and SSS values. Hence, we used total alkalinity (A_T_) from the High Latitude Oceanography Group (GOAL)^79^ and the World Data Center PANGAEA^80^ to estimate A_T_ from SSS using Eq. 1 (*r*^2^ = 0.98, RMSE = 4.4, n = 140).

1 $${\text{A}}_{{\text{T}}} = 36.72 \times {\text{SSS}} + 1052$$

These data were sampled in the austral summers of 1995/96 (PANGAEA; https://doi.pangaea.de/10.1594/PANGAEA.825645;^81^) and 2015–2019 (GOAL; Table S1;^15,22^). Equation 1 was developed using the curve fitting toolbox of MATLAB, with the least absolute residual mode and first-order polynomial adjustment. This option considered all the data important, minimized the residuals, and can be used when data series have few nonconfigurable values^82^. Using the estimated A_T_ and *p*CO_2_^sw^ from SOCATv6, we calculated the total dissolved inorganic carbon (C_T_), pH and saturation states of calcite (Ω_Ca_) and aragonite (Ω_Ar_) with CO_2_SYS version 2.1^83,84^. This program determines these parameters from the thermodynamic equilibrium relation between the carbonate species using carbonate dissociation constants. Because of the good response obtained in high-latitude regions^14, 15,20,85,86^, we used the constants K1 and K2 proposed by Goyet and Poisson^87^ and the sulphate and borate constants proposed by Dickson^88^ and Uppström^89^, respectively.

### Drivers of *p*CO_2_^*sw*^ changes

The *p*CO_2_^sw^ drivers throughout the seasons were calculated based on the difference between the values of the parameters in each season and their respective averages in previous seasons (Δ*p*CO_2_^drv^; Table 1). Then, the Δ*p*CO_2_^drv^ values were separated into categories representing the contributions of differences in C_T_, A_T_, SST and SSS. The relative contributions of the drivers changing *p*CO_2_^sw^ were assessed by converting their relative changes into *p*CO_2_^sw^ units (μatm) following Lenton et al.^55^ as in Eq. 2:

2 $$\Delta p{\text{CO}}_{{2}}^{{{\text{drv}}}} = \frac{{\partial p{\text{CO}}_{2}^{{{\text{sw}}}} }}{{\partial {\text{C}}_{{\text{T}}} }}\Delta {\text{C}}_{{\text{T}}} + \frac{{\partial p{\text{CO}}_{2}^{{{\text{sw}}}} }}{{\partial {\text{A}}_{{\text{T}}} }}\Delta {\text{A}}_{{\text{T}}} + \frac{{\partial p{\text{CO}}_{2}^{{{\text{sw}}}} }}{{\partial {\text{SST}}}}\Delta {\text{SST}} + \frac{{\partial p{\text{CO}}_{2}^{{{\text{sw}}}} }}{{\partial {\text{SSS}}}}\Delta {\text{SSS}}$$

**Table 1:** Average differences (Δ) and standard deviations for the sea surface temperature (SST; °C), salinity (SSS), total alkalinity (A_T_; μmol kg^–1^), and total dissolved inorganic carbon (C_T_; μmol kg^–1^) involved in seawater CO_2_ partial pressure (*p*CO_2_^sw^; μatm) changes.

	Summer	Autumn	Winter	Spring
∆SST	1.47 ± 0.62	− 1.86 ± 0.59	− 0.80 ± 0.34	0.86 ± 0.88
∆SSS	− 0.33 ± 0.48	0.34 ± 0.25	0.25 ± 0.20	− 0.26 ± 0.29
∆A_T_	− 12 ± 18	12 ± 9	9 ± 7	− 9 ± 11
∆C_T_	− 50 ± 52	62 ± 14	25 ± 10	− 36 ± 42
∆*p*CO_2_^drv^	− 69 ± 70	87 ± 24	38 ± 22	− 55 ± 70

The table shows the differences between the values of the parameters in each season and their respective averages in previous seasons (Δ*p*CO_2_^drv^).

where ΔC_T_, ΔA_T_, ΔSST and ΔSSS are the differences between the values of the parameters and their respective averages in previous seasons. This analysis was conducted in each year, and the results were averaged to represent an average year. The partial derivatives were calculated using Eqs. 3 to 6 (see details in Takahashi et al.^3^). These approximations have been widely used in the Southern Ocean^12,21,51^ to evaluate *p*CO_2_^sw^ drivers, both seasonally and spatially. Here, we used the average Revelle and Alkalinity factors of 14 and − 13, respectively.

3 $$\frac{{\partial p{\text{CO}}_{2}^{{{\text{sw}}}} }}{{\partial {\text{C}}_{{\text{T}}} }} = \frac{{p{\text{CO}}_{2}^{{{\text{sw}}}} }}{{{\text{C}}_{{\text{T}}} }} \times {\text{Revelle}}\;{\text{factor}}$$

4 $$\frac{{\partial p{\text{CO}}_{2}^{{{\text{sw}}}} }}{{\partial {\text{A}}_{{\text{T}}} }} = \frac{{p{\text{CO}}_{2}^{{{\text{sw}}}} }}{{{\text{A}}_{{\text{T}}} }} \times {\text{Alkalinity}}\;{\text{factor}}$$

5 $$\frac{{\partial p{\text{CO}}_{2}^{{{\text{sw}}}} }}{{\partial {\text{SSS}}}} \approx 0.026 \times p{\text{CO}}_{2}^{{{\text{sw}}}}$$

6 $$\frac{{\partial p{\text{CO}}_{2}^{{{\text{sw}}}} }}{{\partial {\text{SST}}}}\Delta {\text{SST}} \approx 2 \times p{\text{CO}}_{2}^{{{\text{sw}}}} \times \left( {e^{{0.0423 \times \frac{{\Delta {\text{SST}}}}{2}}} - 1} \right)$$

### Net sea-air CO_2_ flux (FCO_*2*_)

We calculated FCO_2_ using Eq. 7^4, 90^:

7 $${\text{FCO}}_{2} = {\text{K}}_{{\text{t}}} \times {\text{K}}_{{\text{s }}} \times (1 {-} Ice) \Delta p{\text{CO}}_{2 }$$

where ∆*p*CO_2_ is the difference between *p*CO_2_^sw^ and atmospheric *p*CO_2_ (*p*CO_2_^air^); K_t_ is the gas transfer velocity, depending on wind speed^91^; K_s_ is the CO_2_ solubility coefficient, as a function of both SST and SSS^92^; and *Ice* is a dimensionless coefficient corresponding to the fraction of the air–water interface (between 0 and 1) covered by sea ice. We used monthly averages of *p*CO_2_^air^ and wind speed (m s^–1^) data from the U.S. Palmer Station, located in the southern part of the Gerlache Strait. The station continuously measures meteorological parameters throughout the year^68^. We calculated *p*CO_2_^air^ from the monthly averages of the atmospheric molar fraction of CO_2_ (xCO_2_^air^) and atmospheric pressure (both from the Palmer Station), which was corrected by the water vapour pressure estimated from SST and SSS by the widely used equations of Weiss and Price^93^. Sea ice cover was obtained from the monthly mean of the 0.25° daily satellite products by Reynolds et al.^94^, which cover the entire length of the Gerlache Strait (Figure S9e–h).

### Spatial distributions of properties

All spatial distribution maps for the properties in this study were interpolated using Data-Interpolating Variational Analysis (DIVA) gridding^95^. We used a length scale value of 15‰ for both the X and Y axes to ensure optimal preservation of data structure and smoothness. The averaging and all other calculations performed in this study were based only on the observed or reconstructed data and not on the interpolated data. Map interpolations were made to provide reader-friendly visualization of the results.

### Limitations and uncertainties

We estimated the propagated uncertainty from the partial derivatives of all calculated parameters (Table 2) in relation to each variable involved in the calculation as follows:

8 $$\upsigma _{f\left( x \right)} { = }\sqrt {\left( {\frac{\partial f\left( x \right)}{{\partial {\text{variable a}}}}} \right)^{{2}}\upsigma _{{\text{a}}}^{{2}} { + }\left( {\frac{\partial f\left( x \right)}{{\partial {\text{variable b}}}}} \right)^{{2}}\upsigma _{{\text{b}}}^{{2}} { + } \cdots { + }\left( {\frac{\partial f\left( x \right)}{{\partial {\text{variable z}}}}} \right)^{{2}}\upsigma _{{\text{z}}}^{{2}} { }}$$

**Table 2:** Average and standard deviation of the uncertainties propagated in the calculations of the carbonate system properties and net sea-air CO_2_ flux (FCO_2_) for each season.

	*p*CO_2_^sw^ uncertainty	Summer	Autumn	Winter	Spring
FCO_2_	< 2 µatm (55% of all data)	1.96 ± 0.64	2.65 ± 0.83	3.46 ± 1.18	2.34 ± 0.76
C_T_		5.58 ± 0.57	5.00 ± 0.10	4.84 ± 0.05	5.13 ± 0.46
Ω_Ca_		0.15 ± 0.03	0.12 ± 0.01	0.11 ± 0.003	0.12 ± 0.03
Ω_Ar_		0.10 ± 0.02	0.07 ± ~ 0	0.07 ± ~ 0	0.08 ± 0.02
pH		0.0076 ± 0.0003	0.0075 ± 0.0003	0.0075 ± ~ 0	0.0075 ± 0.0002
FCO_2_	< 5 µatm (45% of all data)	4.15 ± 0.73	6.53 ± 2.10	8.29 ± 3.04	5.44 ± 1.80
C_T_		6.11 ± 1.16	5.22 ± 0.13	5.02 ± 0.07	5.45 ± 0.83
Ω_Ca_		0.16 ± 0.04	0.12 ± 0.01	0.11 ± 0.003	0.13 ± 0.03
Ω_Ar_		0.10 ± 0.02	0.07 ± ~ 0	0.07 ± ~ 0	0.08 ± 0.02
pH		0.0097 ± 0.0015	0.0087 ± ~ 0	0.0086 ± 0.0001	0.0090 ± 0.0010

The units of uncertainty are the same as the units of the evaluated parameters: FCO_2_ (mmol m^–2^ day^–1^), total dissolved inorganic carbon (C_T_; μmol kg^–1^), pH (total scale) and saturation states of calcite (Ω_Ca_) and aragonite (Ω_Ar_) (unitless). Standard deviations ~ 0 are smaller than the limit of significant digits in the averages.

where the derived functions $f(x)$ are the calculated parameters (i.e., FCO_2_, C_T_, Ω and pH) and σ is the uncertainty associated with each variable involved in calculation of the parameter. Because SSS uncertainties are expected to be low enough to be negligible (i.e., < 0.001, according to the GOAL and PANGAEA datasets), they were not considered here. Hence, the propagated uncertainties in C_T_, Ω and pH fundamentally represented the errors associated with the estimated A_T_ (± 4.4 μmol kg^–1^), SST (± 0.05 °C) and measured *p*CO_2_^sw^. We used *p*CO_2_^sw^ data from SOCATv6 with uncertainties < 2 µatm (55% of total) and < 5 µatm (45%). We calculated the propagated uncertainties for all carbonate system properties with the CO_2_SYS error tool^96^. For FCO_2_, uncertainty was related to the standard error of the averaged wind speed for each season, the measured *p*CO_2_^sw^ and xCO_2_^air^, and sea ice cover. The analytical error for xCO_2_^air^ measurements from the U.S. Palmer Station was estimated to be ± 0.07 µmol/mol for the studied period^68^. Sea ice concentrations were computed to a precision of 1% coverage^94,97^.

Finally, we used a first-order polynomial relationship between A_T_ and SSS to estimate A_T_ and calculate the other parameters of the carbonate system based on summertime data. We assumed this relationship for all seasons because the summer was the only period with available A_T_ data for the study region. However, the summer is characterized by greater A_T_ variability than other seasons, implying that the ranges of A_T_ and SSS may represent the annual range (i.e., A_T_: 2200–2320 μmol kg^−1^; SSS: 32–34.5). Such limitations are mainly due to the scarcity of data in periods other than summer and highlight the need for additional efforts to better understand the dynamics of the carbonate system parameters in coastal regions of the Southern Ocean.

## Supplementary information

Supplementary file1
