# Author Correction: Seasonal variability of net sea-air CO_2_ fluxes in a coastal region of the northern Antarctic Peninsula

**DOI:** 10.1038/s41598-020-79111-6

**Published:** 2020-12-09

**Authors:** Thiago Monteiro, Rodrigo Kerr, Eunice da Costa Machado

**Affiliations:** 1 Programa de Pós-Graduação em Oceanologia, Instituto de Oceanografia, Universidade Federal do Rio Grande (FURG), Av. Itália km 8, Rio Grande, RS 96203-900 Brazil; 2 Laboratório de Estudos dos Oceanos e Clima, Instituto de Oceanografia, FURG, Rio Grande, RS Brazil; 3 Brazilian Ocean Acidification Network (BrOA), Rio Grande, RS Brazil; 4 Laboratório de Hidroquímica, Instituto de Oceanografia, FURG, Rio Grande, RS Brazil

Correction to: *Scientific Reports* 10.1038/s41598-020-71814-0, published online 10 September 2020

This Article contains an error in the y-axis label of Figure 5, where the unit of measurement is incorrect.

“FCO_2_ (mmol m^-1^ day^-1^)” should read: “FCO_2_ (mmol m^-2^ day^-1^)”

The correct Figure 5 appears below as Figure 1.

**Figure 1:**
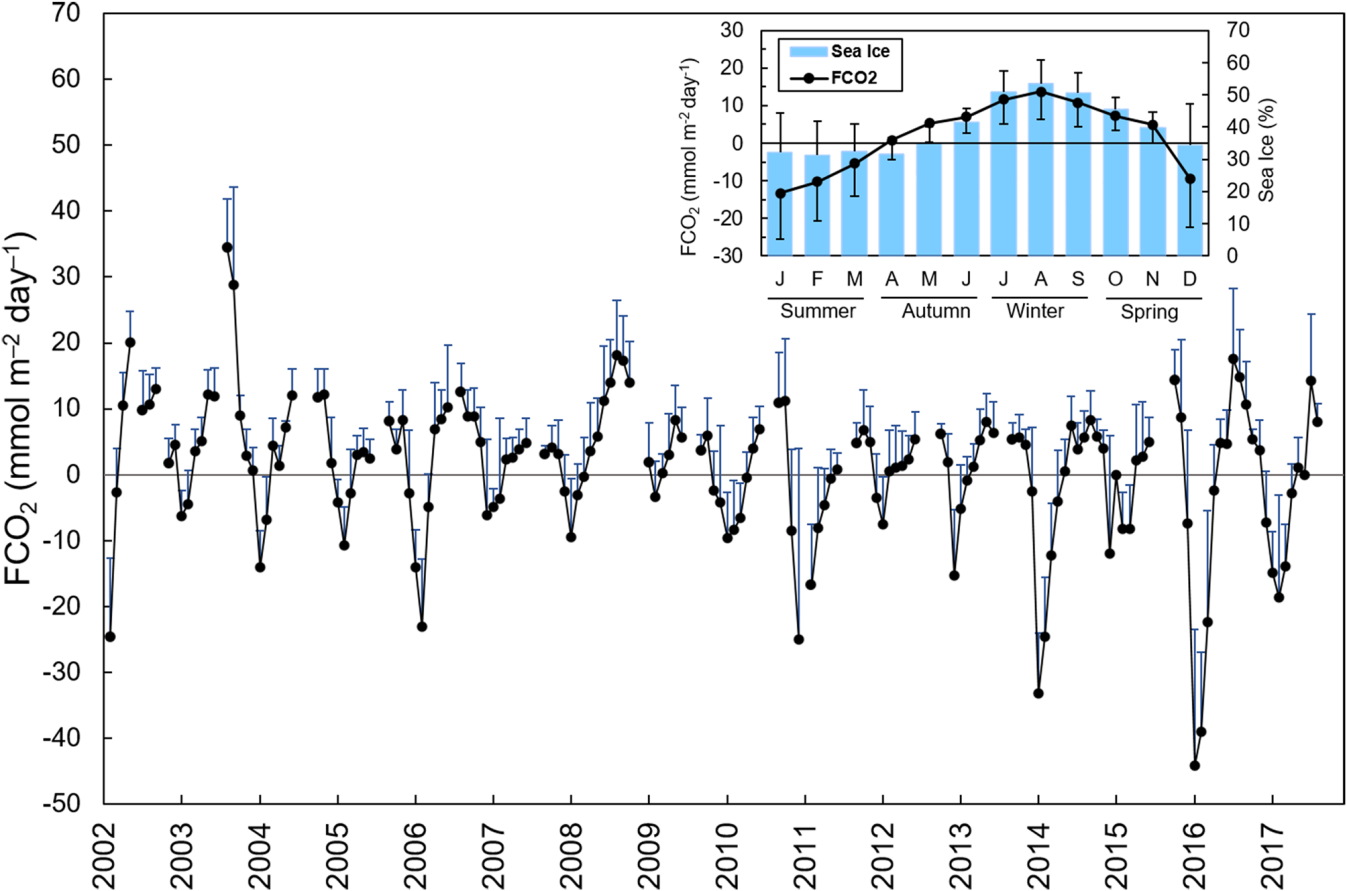
A correct version of the original Figure 5**.** Monthly averages of net sea-air CO_2_ fluxes (FCO_2_) in the Gerlache Strait from January 2002 to December 2017 with an inset showing the variability throughout the year to characterize the seasonal cycle of FCO_2_ and the percentage of sea ice cover (filled blue bars). The gaps are from years when there was no winter sampling in the region. The blue bars oriented upwards are the standard deviations from the respective monthly averages, as are the black bars in the inset. Positive FCO_2_ values represent the outgassing of CO_2_ to the atmosphere, whereas negative FCO_2_ values represent CO_2_ uptake by the ocean.

