# Supplementary material for: Seasonal variability of net sea-air CO2 fluxes in a coastal region of the northern Antarctic Peninsula
Source: Sci Rep. 2020 Sep 10;10:14875. doi: 10.1038/s41598-020-71814-0 (PMC7483740; doi:10.1038/s41598-020-71814-0)
Supplement: Supplementary file 1 — Supplementary file1 [file 41598_2020_71814_MOESM1_ESM.docx]

**Seasonal variability of net sea-air CO_2_ fluxes in a coastal region of the northern Antarctic Peninsula**

Thiago Monteiro^1,2,3^, Rodrigo Kerr^1,2,3^ and Eunice Machado^1,4^

^1^Programa de Pós-Graduação em Oceanologia, Instituto de Oceanografia, Universidade Federal do Rio Grande (FURG), Av. Itália km 8, Rio Grande, 96203-900, RS, Brazil.

^2^Laboratório de Estudos dos Oceanos e Clima, Instituto de Oceanografia, Universidade Federal do Rio Grande (FURG), Av. Itália km 8, Rio Grande, 96203-900, RS, Brazil.

^3^Brazilian Ocean Acidification Network (BrOA), Rio Grande, 96203-900, RS, Brazil.

^4^Laboratório de Hidroquímica, Instituto de Oceanografia, Universidade Federal do Rio Grande (FURG), Av. Itália km 8, Rio Grande, 96203-900, RS, Brazil.

Supplementary information

The file contains:

Text S1: Sea ice growth and melt processes.

Text S2: Normalization of A_T_ and C_T_ by average seasonal salinity.

Text S3: Meteoric water percentage.

Figure S1: End-members of A_T_ and C_T_ used to estimate the theoretical line of the sea ice growth/sea ice melt processes.

Figure S2: Data coverage per year in each season available for the Gerlache Strait from SOCATv6.

Figure S3: Data distribution in each season with the percentages within each month.

Figure S4: Surface distributions of temperature and salinity throughout the seasons.

Figure S5: Surface distributions of AT and CT throughout the seasons.

Figure S6: Surface distributions of pCO_2_^sw^ and ΔpCO_2_ throughout the seasons.

Figure S7: Surface distributions of ΩCa and ΩAr throughout the seasons.

Figure S8: Surface distribution of pH throughout the seasons.

Figure S9: Surface distributions of meteoric water and sea ice cover percentage throughout the seasons.

Figure S10: Linear correlation between the seasonal cycle of FCO_2_ and the percentage sea ice cover in the Gerlache Strait.

Table S1: GOAL dataset from 2015 to 2019 used to build the relationship between total alkalinity (A_T_) and sea surface salinity (SSS).

*Text S1: Sea ice growth and melt processes*

The sea ice growth and sea ice melt processes represented by the theoretical grey arrow in Figure 3 were built based on the end-members of total alkalinity (A_T_) and total dissolved inorganic carbon (C_T_) described in Rysgaard et al. (2011) [1] and references therein. For sea ice growth, the authors estimated that the salinity, A_T_ and C_T_ values were 33.98, 2361 µmol kg^-1^ and 2219 µmol kg^-1^, respectively, and for sea ice melt, these values were 6, 864 µmol kg^-1^ and 480 µmol kg^-1^, respectively. Therefore, we adapted the widely known biogeochemical diagram (e.g., Zeebe, 2012 [2]; Wanninkhof et al., 2015 [3]) and included these two processes through the theoretical line in Figure S1. Therefore, the closer the relationship between nA_T_ and nC_T_ is to this theoretical line, the more likely it will be that the sea ice growth/sea ice melt processes are influencing nA_T_ and nC_T_.


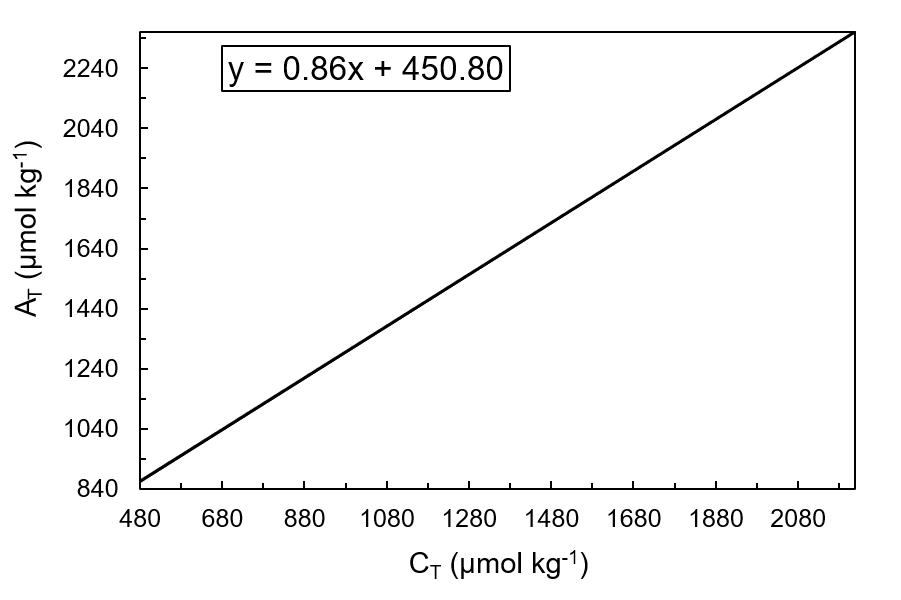


Figure S1: End-members used in the construction of the theoretical line of the sea ice growth/sea ice melt processes (grey arrow) in Figure 3. This line was based on the A_T_ and C_T_ values for meltwater (864 µmol kg^-1^ and 480 µmol kg^-1^, respectively) and for sea ice growth (2361 µmol kg^-1^ and 2219 µmol kg^-1^, respectively), as suggested by Rysgaard et al. (2011) [1] and references therein.

*Text S2: Normalization of A_T_ and C_T_ by average seasonal salinity*

A_T_ and C_T_ data were normalized to a seasonal average salinity using a non-zero freshwater end-member according to the equation by Friis et al. (2003) [4]:

$$nA_{T}\text{ = }\left. \frac{A_{\text{T}}^{\text{meas}}-A_{\text{T}}^{\text{s=0}}}{\mathrm{SSS}^{\mathrm{meas}}} \right.\text{ × }\mathrm{SSS}^{\mathrm{avg}}+A_{\text{T}}^{\text{s=0}}$$

where A_T_^meas^ is the measured A_T_, A_T_^s=0^ is the A_T_ for a non-zero salinity, SSS^meas^ is the measured salinity and SSS^avg^ is the average salinity for each season. The same equation was used to calculate the C_T_ normalized by salinity. We used A_T_^S=0^ = 864 µmol kg^-1^ and C_T_^S=0^ = 480 µmol kg^-1^ from the end-members proposed by Rysgaard et al. (2011) [1] and references therein.

*Text S3: Meteoric water*

We estimated the meteoric water percentage (MW) using Eq. (S1), following Rivaro et al. (2011) [5], which is widely used in polar regions (e.g., Rivaro et al., 2014 [6]; Mendes et al., 2018 [7]), and assuming an average sea-ice salinity of 6 (Ackley et al. 1979 [8]):

$$\text{Meteoric Water}\text{ }\text{=}\left( \text{1-}\frac{\text{Salinty}_{\text{surface}}-6}{\text{Salinity}_{\text{botton}}-6} \right)\text{×100}$$

where S_surface_ and S_botton_ are the salinities at the surface and bottom of the ocean, respectively. Here, we considered an average bottom salinity of 34.50 following Monteiro et al. (2020) [9], who estimated it from the GOAL and World Ocean Database 2013 (WOD13) datasets. MW was defined by Meredith et al. (2008) [10] as the freshwater contribution without source distinction from precipitation, glacial discharge and iceberg or sea ice melt.


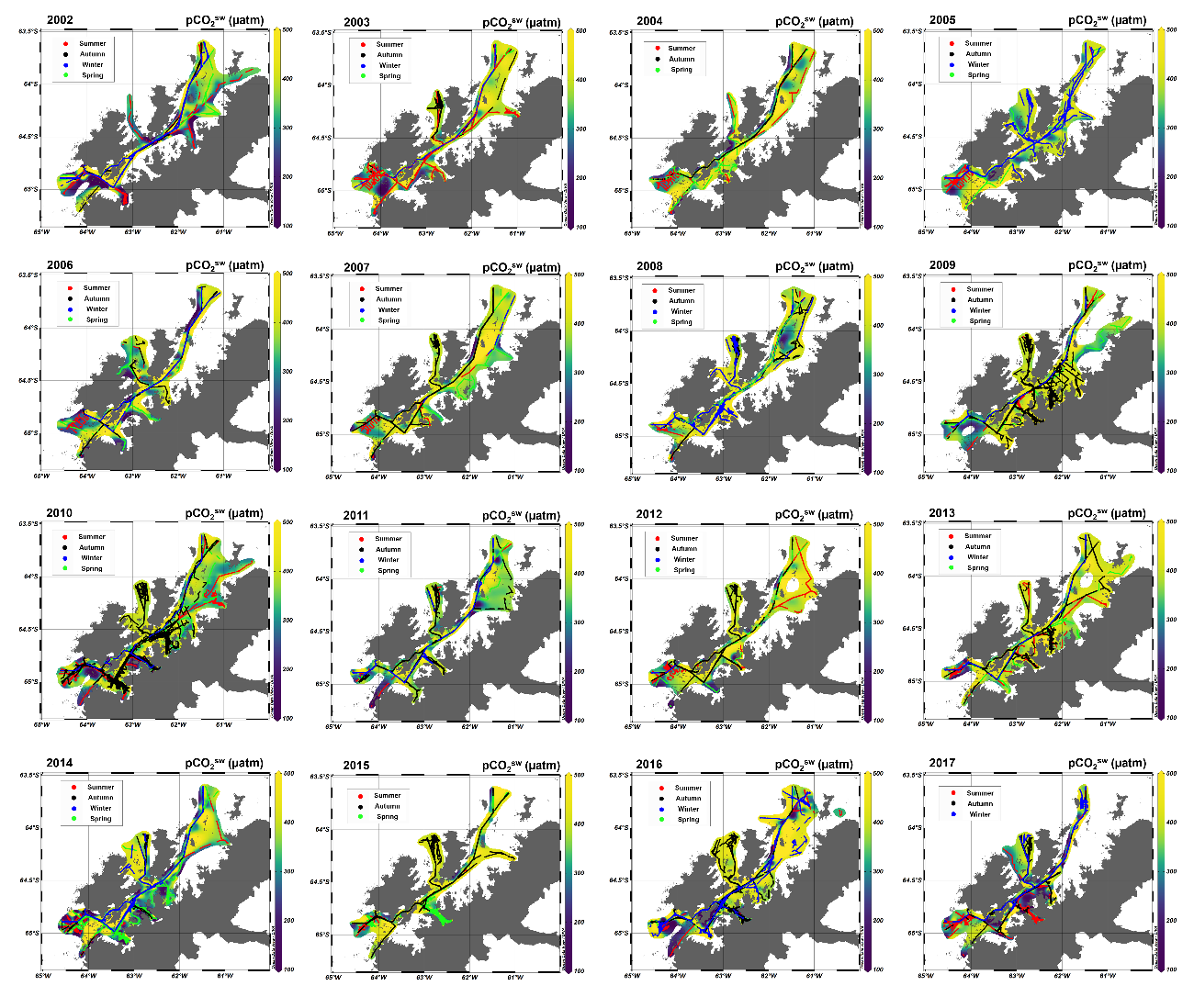


Figure S2: Data coverage of pCO_2_^sw^ (with temperature and salinity) per year in each season available for the Gerlache Strait from SOCATv6. The coloured circles indicate the seasons. The percentage of data in each month of the seasons is shown in Figure S3. These maps were generated by using the software Ocean Data View (v. 5.3.0, <http://odv.awi.de>) [11].


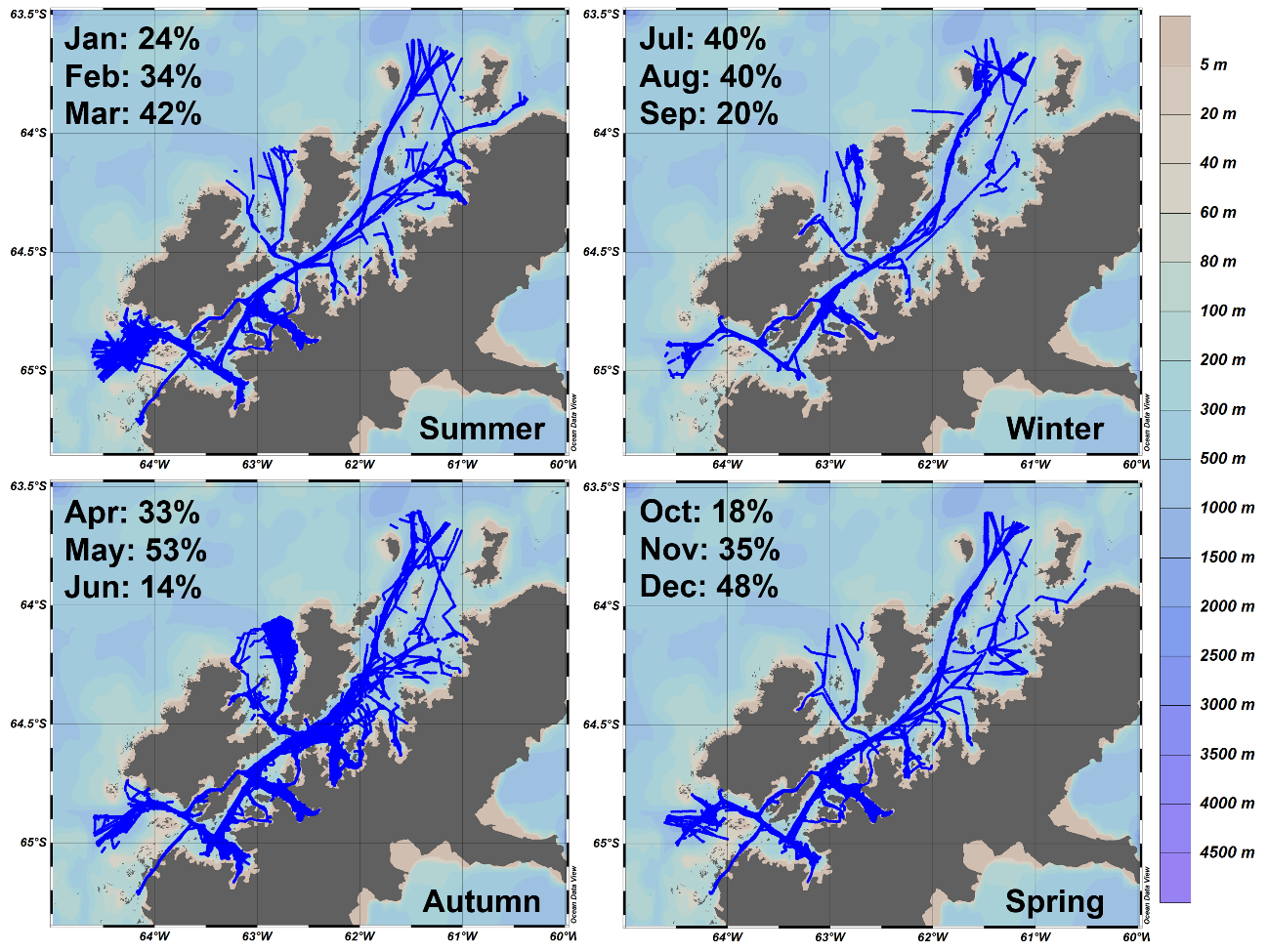


Figure S3: Density and distribution of data in each season with the data percentages within each month. At each point, temperature, salinity and pCO_2_^sw^ data were measured and used to derive the other parameters. These maps were generated by using the software Ocean Data View (v. 5.3.0, <http://odv.awi.de>) [11].


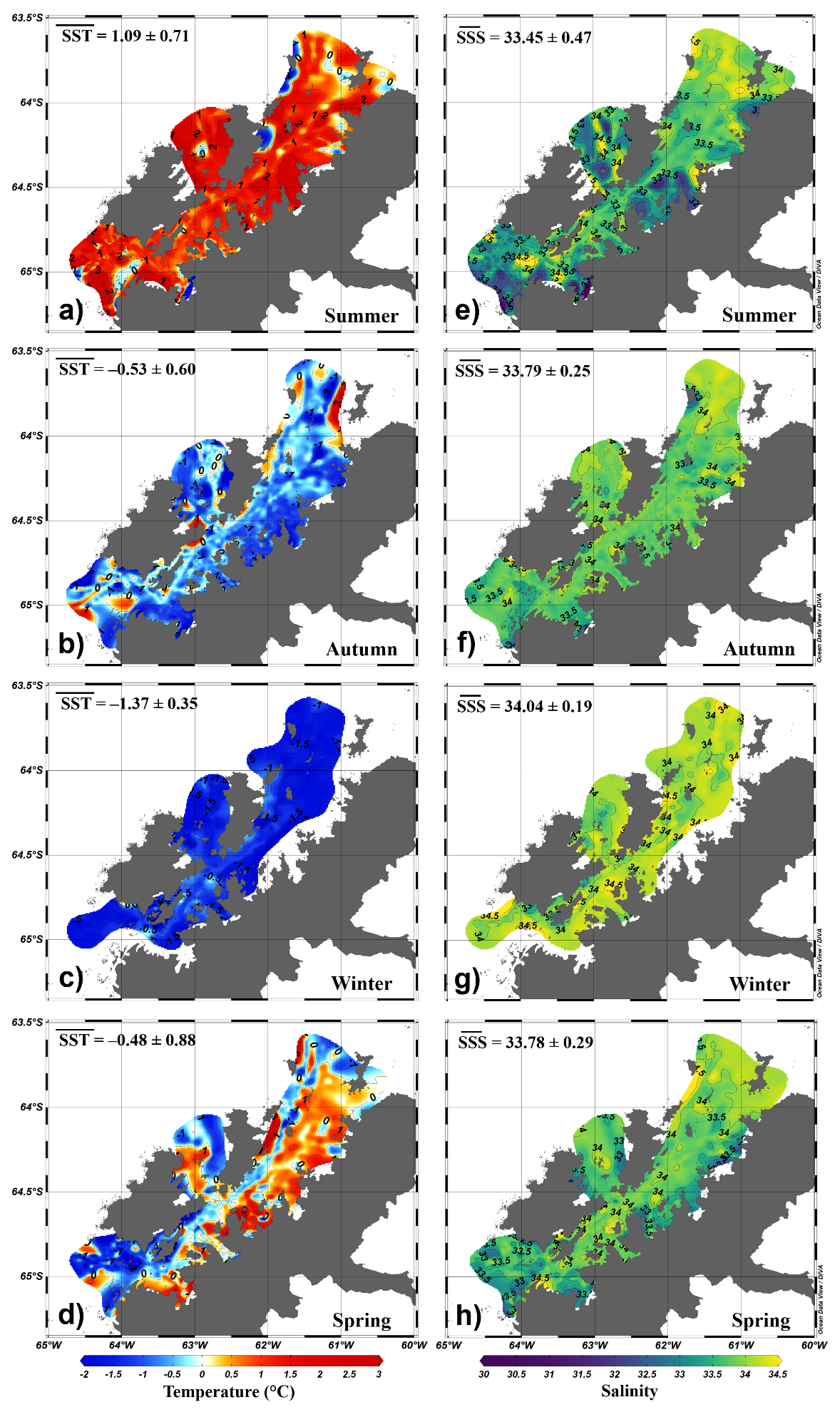


Figure S4: Seasonal surface distribution of temperature (a-d) and salinity (e-h) in the Gerlache Strait from detrended time series from 2002 to 2017. The values indicate the average and standard deviation of each property for the entire area and season. These maps were generated by using the software Ocean Data View (v. 5.3.0, <http://odv.awi.de>) [11].


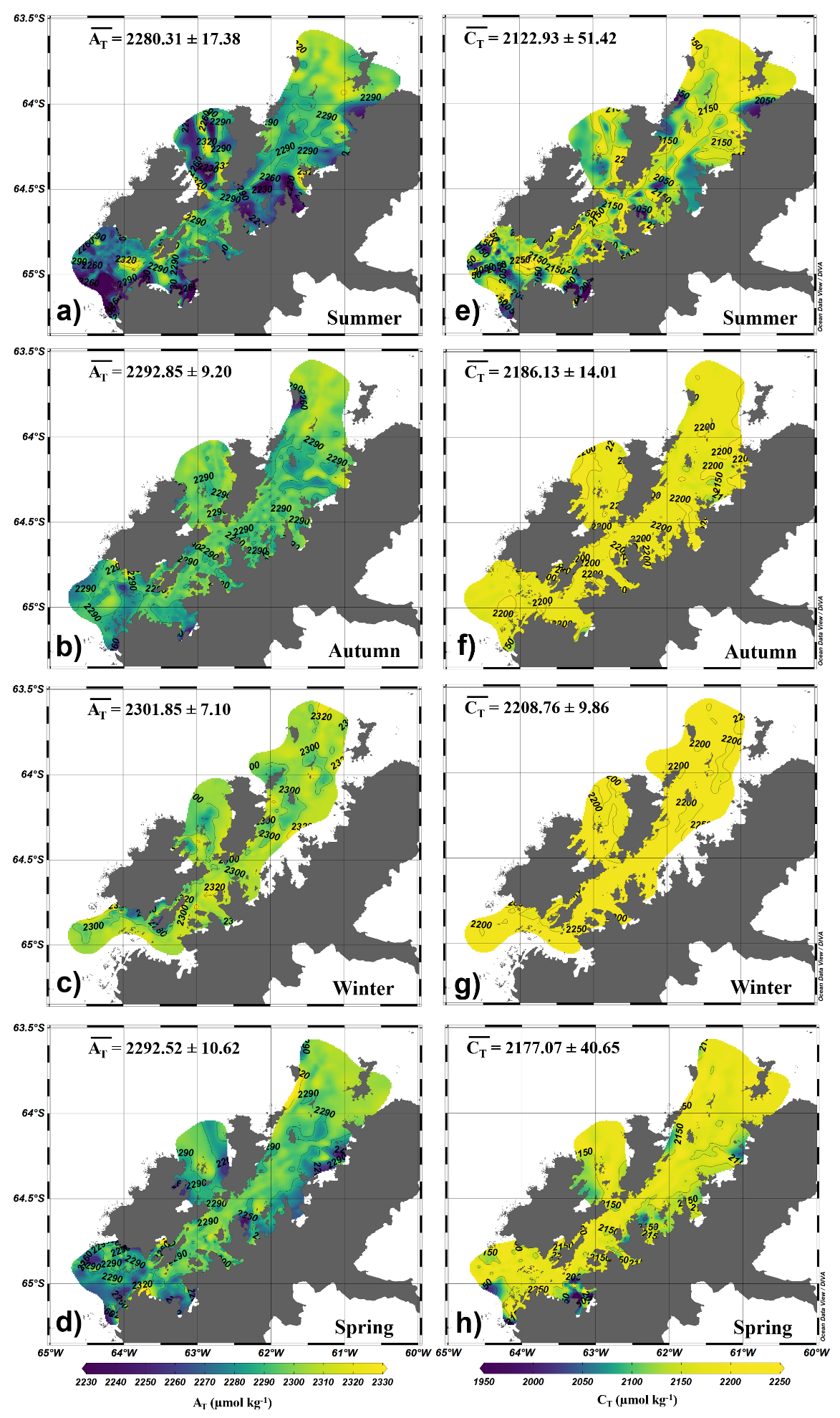


Figure S5: Same as Figure S2 except for total alkalinity–A_T_ (a-d) and total dissolved inorganic carbon–C_T_ (e-h). These maps were generated by using the software Ocean Data View (v. 5.3.0, <http://odv.awi.de>) [11].


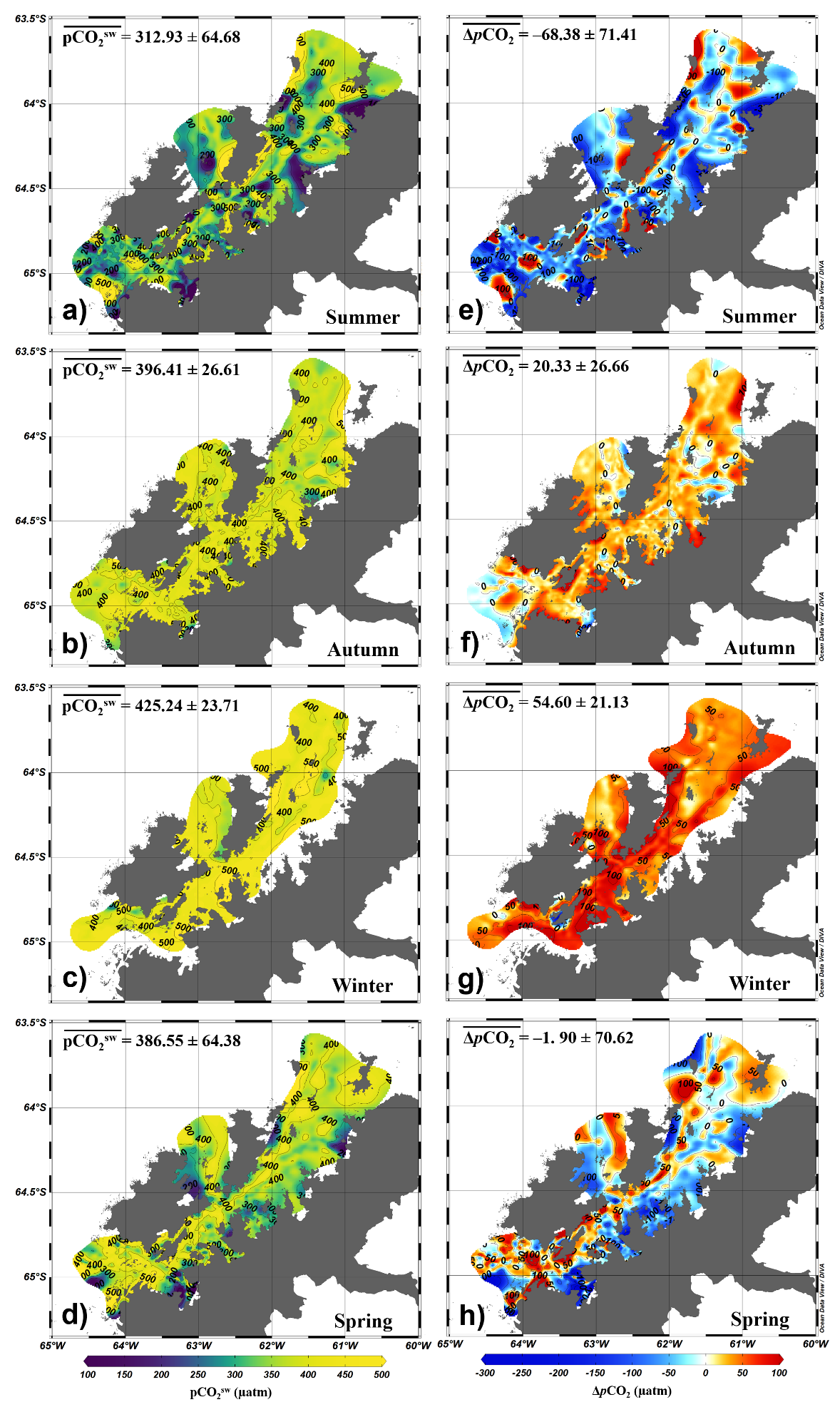


Figure S6: Same as Figure S2 except for pCO_2_^sw^ (a-d) and ΔpCO2 (pCO_2_^sw^ – pCO_2_^air^) (e-h). These maps were generated by using the software Ocean Data View (v. 5.3.0, <http://odv.awi.de>) [11].


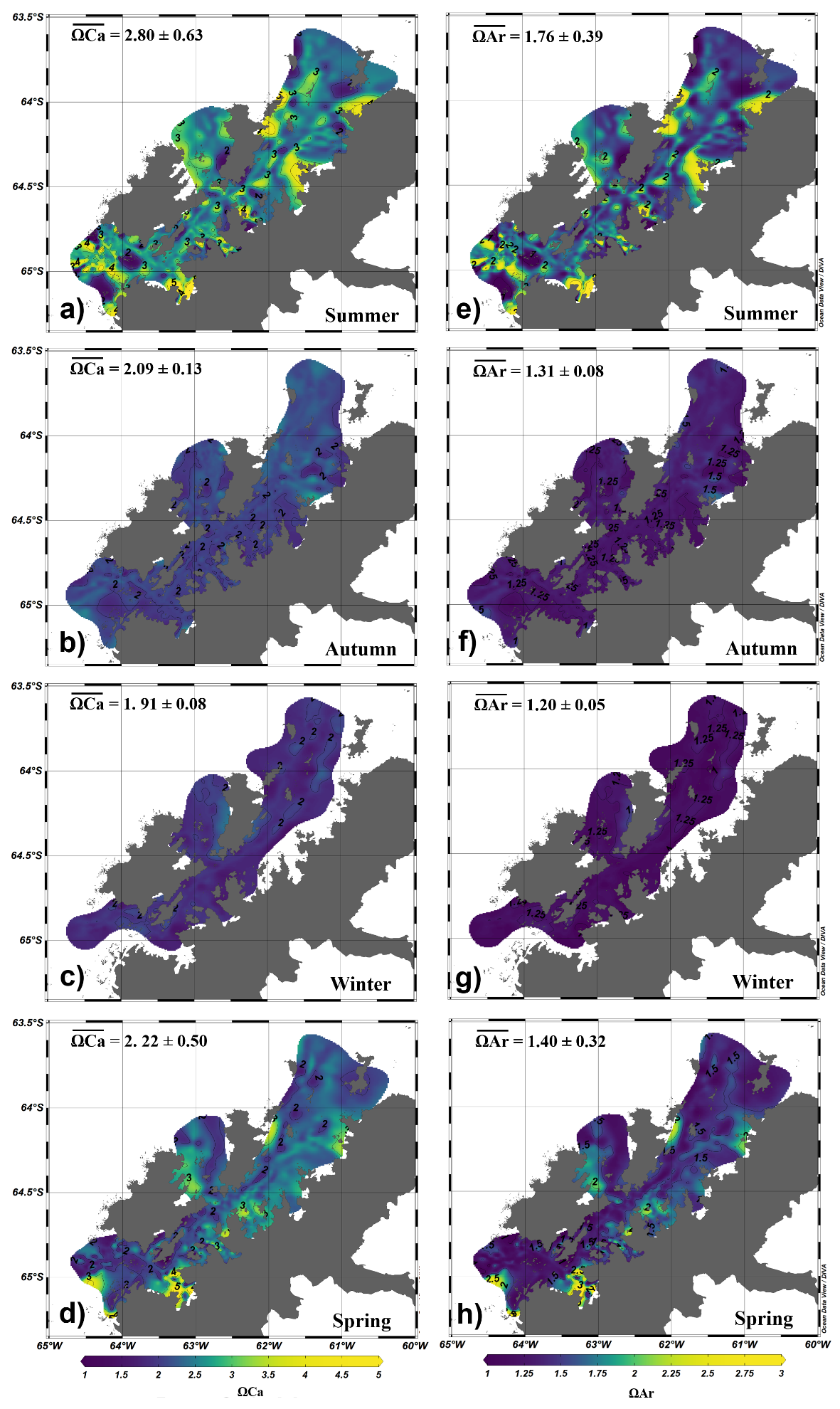


Figure S7: Same as Figure S2 except for the saturation states of calcite–ΩCa (a-d) and aragonite–ΩAr (e-h). These maps were generated by using the software Ocean Data View (v. 5.3.0, <http://odv.awi.de>) [11].


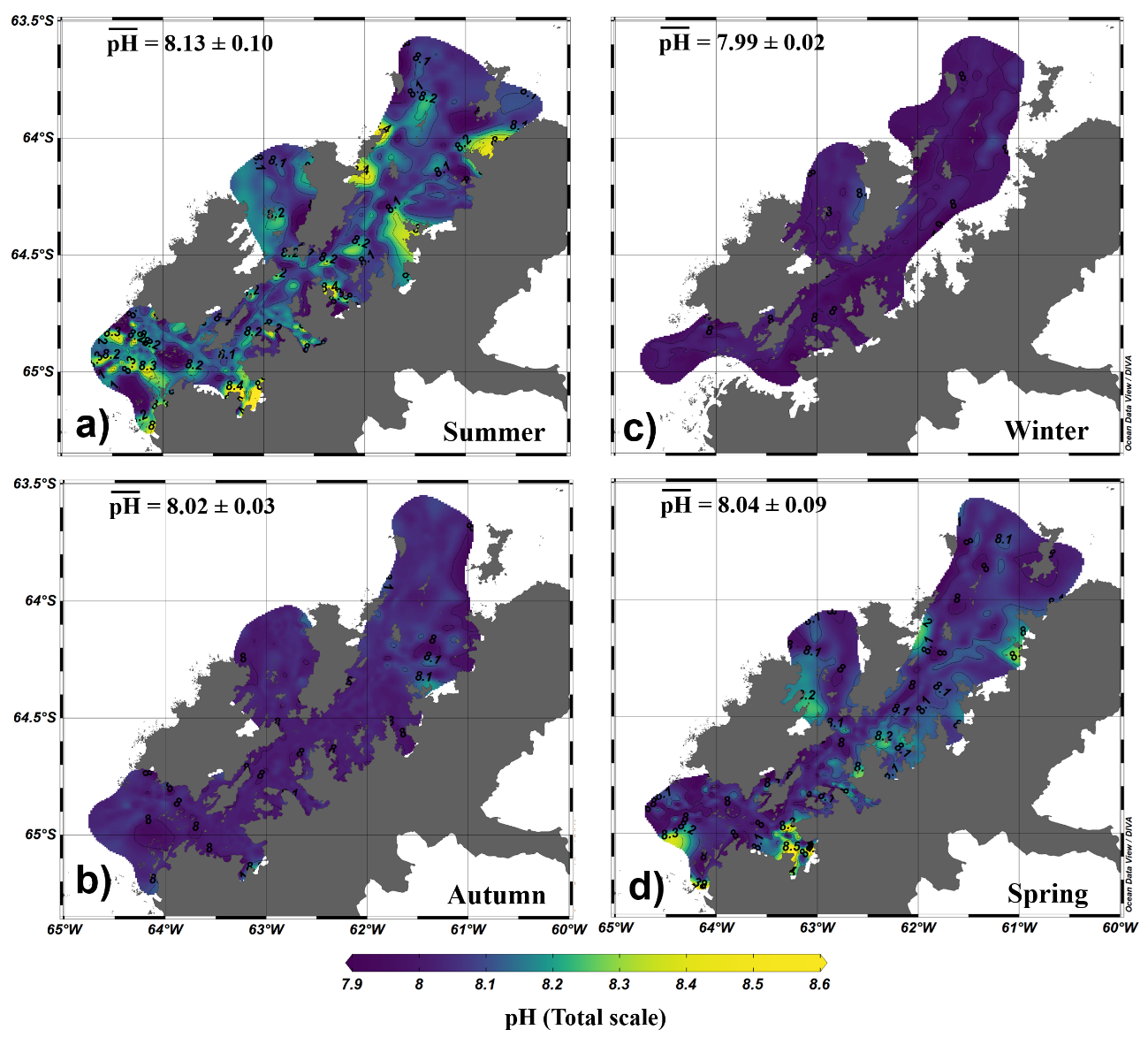


Figure S8: Same as Figure S2 except for pH. These maps were generated by using the software Ocean Data View (v. 5.3.0, <http://odv.awi.de>) [11].


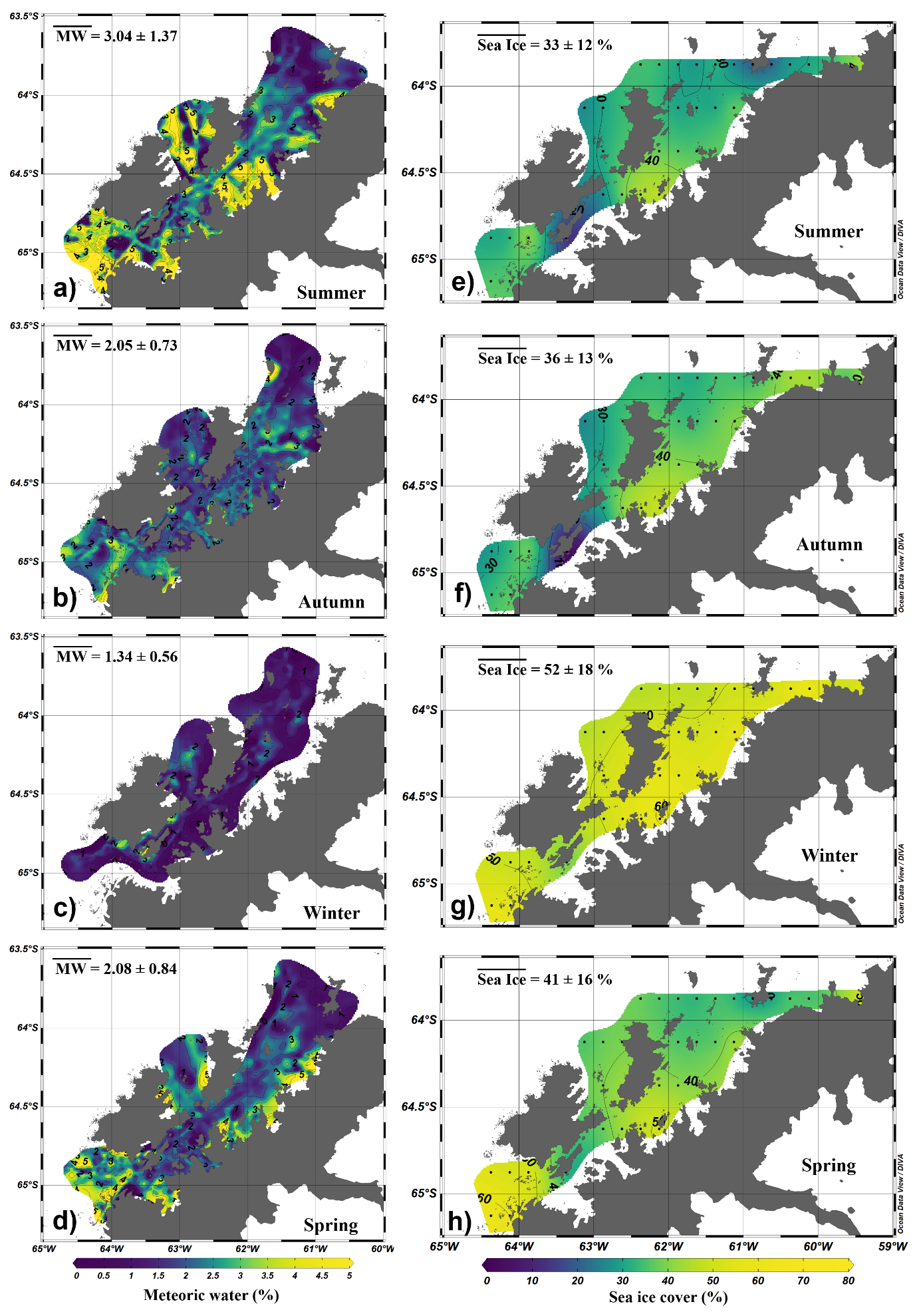


Figure S9: Same as Figure S2 except for meteoric water (a-d) and sea ice cover (e-h) percentage. These maps were generated by using the software Ocean Data View (v. 5.3.0, <http://odv.awi.de>) [11].


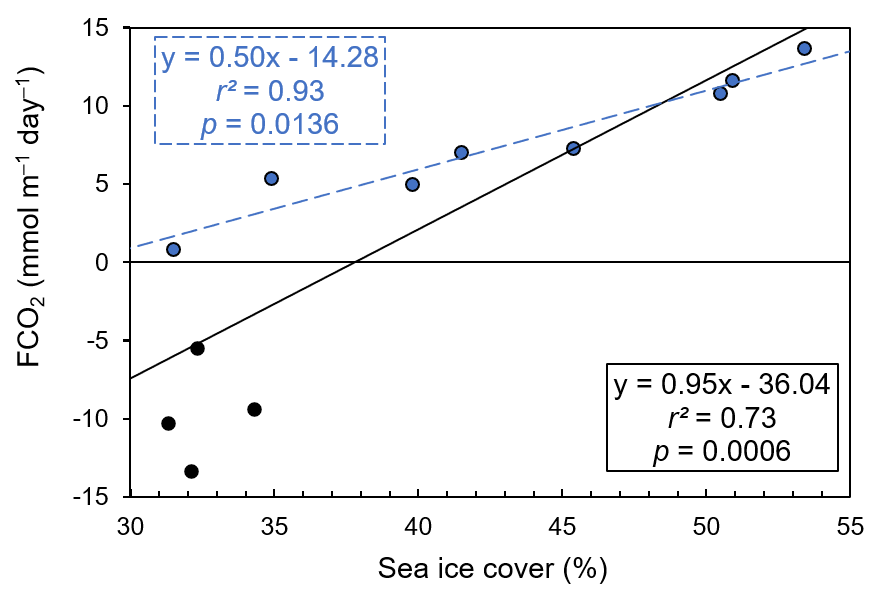


Figure S10: Linear correlation between the seasonal cycle of the net sea-air CO_2_ flux (FCO2) and the percentage of sea ice cover in the Gerlache Strait. Each point represents the monthly averaged values of FCO2 and sea ice cover between 2002 and 2017. The sea ice data are from the 0.25° daily satellite products from Reynolds et al. (2007) [12]. The continuous black line represents the linear fit for all months of the year, and its statistical properties are shown in the continuous black rectangle. The dashed line represents the linear fit only for the months in which the region exhibits CO_2_ outgassing (i.e., from April to November; blue-filled circles), and its statistical properties are shown in the dashed blue rectangle.

Table S1: GOAL dataset from 2015 to 2019 used to build the relationship between total alkalinity (A_T_) and sea surface salinity (SSS). In all these years, A_T_, SSS, temperature (SST) and total dissolved inorganic carbon (C_T_) were measured/analysed onboard the Brazilian Navy RV Almirante Maximiano.

| Cruise | **Date** | **hh:mm** | **Longitude** | **Latitude** | **Depth (m)** | **SST (°C)** | **SSS** | **AT  (umol kg^-1^)** | **CT  (umol kg^-1^)** |
| --- | --- | --- | --- | --- | --- | --- | --- | --- | --- |
| Nautilus I | 2/8/2015 | 22:21 | -63.356 | -64.955 | 6 | 0.855 | 33.328 | 2276.60 | 2151.85 |
| Nautilus I | 2/8/2015 | 23:44 | -63.199 | -64.836 | 5 | 0.698 | 33.587 | 2253.75 | 2162.37 |
| Nautilus I | 2/8/2015 | 8:50 | -61.390 | -63.719 | 6 | 0.727 | 34.263 | 2399.07 | 2186.22 |
| Nautilus I | 2/9/2015 | 1:22 | -63.000 | -64.665 | 6 | 0.820 | 33.595 | 2250.88 | 2173.61 |
| Nautilus I | 2/9/2015 | 3:50 | -62.506 | -64.569 | 5 | 0.775 | 33.648 | 2264.47 | 2174.80 |
| Nautilus I | 2/9/2015 | 7:00 | -62.115 | -64.428 | 5 | 1.507 | 33.532 | 2273.35 | 2166.91 |
| Nautilus I | 2/9/2015 | 13:20 | -62.909 | -64.464 | 5 | 1.417 | 33.530 | 2294.34 | 2178.83 |
| Nautilus I | 2/9/2015 | 15:33 | -63.004 | -64.385 | 5 | 1.806 | 33.610 | 2289.10 | 2132.37 |
| Nautilus I | 2/9/2015 | 17:25 | -62.767 | -64.520 | 5 | 1.107 | 33.711 | 2279.57 | 2192.52 |
| Nautilus I | 2/9/2015 | 20:48 | -61.830 | -64.337 | 5 | 1.513 | 33.666 | 2298.22 | 2248.51 |
| Nautilus I | 2/9/2015 | 22:22 | -61.732 | -64.376 | 5 | 1.231 | 33.728 | 2285.74 | 2212.22 |
| Nautilus I | 2/9/2015 | 23:50 | -61.572 | -64.248 | 6 | 0.983 | 33.683 | 2272.28 | 2225.59 |
| Nautilus I | 2/10/2015 | 1:47 | -61.397 | -64.170 | 6 | 1.580 | 33.617 | 2291.33 | 2195.91 |
| Nautilus I | 2/10/2015 | 7:17 | -60.780 | -63.949 | 5 | 0.333 | 34.199 | 2297.50 | 2216.12 |
| Nautilus II | 2/15/2016 | 7:09 | -63.362 | -64.954 | 5 | 0.641 | 33.871 | 2318.05 | 2142.02 |
| Nautilus II | 2/15/2016 | 8:55 | -63.209 | -64.840 | 5 | 0.586 | 33.908 | 2317.68 | 2146.00 |
| Nautilus II | 2/15/2016 | 11:04 | -63.002 | -64.667 | 5 | 0.543 | 34.036 | 2314.22 | 2153.87 |
| Nautilus II | 2/14/2016 | 22:29 | -63.003 | -64.385 | 5 | 1.080 | 33.910 | 2306.24 | 2177.40 |
| Nautilus II | 2/14/2016 | 23:54 | -62.907 | -64.466 | 6 | 0.910 | 33.951 | 2309.54 | 2177.31 |
| Nautilus II | 2/15/2016 | 2:27 | -62.763 | -64.520 | 5 | 0.681 | 33.972 | 2331.62 | 2185.68 |
| Nautilus II | 2/15/2016 | 13:02 | -62.507 | -64.570 | 5 | 0.596 | 33.945 | 2325.30 | 2153.64 |
| Nautilus II | 2/15/2016 | 18:15 | -61.931 | -64.306 | 5 | 0.942 | 33.849 | 2333.94 | 2072.34 |
| Nautilus II | 2/15/2016 | 20:15 | -61.829 | -64.335 | 5 | 0.895 | 33.864 | 2318.03 | 2083.93 |
| Nautilus II | 2/15/2016 | 22:11 | -61.736 | -64.374 | 6 | 0.892 | 33.855 | 2325.90 | 2059.60 |
| Nautilus II | 2/16/2016 | 1:33 | -61.399 | -64.165 | 5 | 1.017 | 34.049 | 2355.33 | 2089.25 |
| Nautilus II | 2/16/2016 | 5:13 | -60.785 | -63.951 | 6 | -0.139 | 34.335 | 2343.45 | 2203.58 |
| Nautilus III | 2/14/2017 | 22:48 | -63.359 | -64.954 | 5 | 2.229 | 33.499 | 2297.77 | 2142.56 |
| Nautilus III | 2/15/2017 | 19:43 | -63.202 | -64.837 | 5 | 1.785 | 33.766 | 2298.88 | 2107.21 |
| Nautilus III | 2/15/2017 | 21.52 | -63.001 | -64.666 | 5 | 1.450 | 33.852 | 2288.09 | 2113.60 |
| Nautilus III | 2/16/2017 | 3:43 | -63.005 | -64.385 | 5 | 2.999 | 33.518 | 2320.05 | 2123.49 |
| Nautilus III | 2/16/2017 | 2:07 | -62.908 | -64.466 | 7 | 1.806 | 33.820 | 2349.49 | 2187.67 |
| Nautilus III | 2/16/2017 | 0:46 | -62.761 | -64.520 | 4 | 2.749 | 33.695 | 2387.66 | 2288.62 |
| Nautilus III | 2/16/2017 | 7:51 | -62.507 | -64.570 | 6 | 1.554 | 33.524 | 2363.38 | 2286.14 |
| Nautilus III | 2/16/2017 | 12:30 | -62.105 | -64.425 | 5 | 1.281 | 33.827 | 2305.98 | 2133.50 |
| Nautilus III | 2/16/2017 | 17:32 | -61.927 | -64.303 | 5 | 2.368 | 33.418 | 2313.12 | 2099.42 |
| Nautilus III | 2/16/2017 | 16:06 | -61.827 | -64.336 | 5 | 2.236 | 33.408 | 2308.40 | 2092.59 |
| Nautilus III | 2/16/2017 | 14:39 | -61.747 | -64.375 | 5 | 2.371 | 33.221 | 2280.42 | 2021.94 |
| Nautilus III | 2/16/2017 | 21:11 | -61.591 | -64.230 | 5 | 2.439 | 33.312 | 2304.18 | 2093.57 |
| Nautilus III | 2/16/2017 | 22:41 | -61.407 | -64.469 | 5 | 1.498 | 33.830 | 2296.64 | 2091.35 |
| Nautilus_IV | 2/21/2018 | 4:44 | -61.393 | -63.725 | 7 | 0.590 | 34.285 | 2387.67 | 2278.70 |
| Nautilus_IV | 2/21/2018 | 9:52 | -60.779 | -63.955 | 6 | 0.390 | 34.161 | 2325.72 | 2233.44 |
| Nautilus_IV | 2/21/2018 | 13:05 | -61.417 | -64.163 | 5 | 1.846 | 33.576 | 2298.19 | 2107.17 |
| Nautilus_IV | 2/21/2018 | 15:34 | -61.590 | -64.233 | 6 | 1.461 | 33.653 | 2279.27 | 2164.64 |
| Nautilus_IV | 2/21/2018 | 21:21 | -61.740 | -64.377 | 6 | 1.418 | 33.655 | 2309.39 | 2168.37 |
| Nautilus_IV | 2/21/2018 | 19:43 | -61.817 | -64.334 | 5 | 1.529 | 33.617 | 2319.07 | 2155.82 |
| Nautilus_IV | 2/21/2018 | 23:37 | -62.126 | -64.427 | 5 | 1.255 | 33.686 | 2311.97 | 2185.73 |
| Nautilus_IV | 2/21/2018 | 17:43 | -61.939 | -64.305 | 6 | 1.752 | 33.526 | 2298.04 | 2131.49 |
| Nautilus_IV | 2/22/2018 | 10:57 | -62.502 | -64.570 | 9 | 1.079 | 33.803 | 2317.38 | 2213.98 |
| Nautilus_IV | 2/22/2018 | 19:35 | -62.764 | -64.520 | 7 | 1.778 | 33.772 | 2323.92 | 2193.94 |
| Nautilus_IV | 2/22/2018 | 15:21 | -63.003 | -64.387 | 5 | 1.727 | 33.656 | 2320.95 | 2189.67 |
| Nautilus_IV | 2/22/2018 | 17:32 | -62.903 | -64.465 | 5 | 1.890 | 33.834 | 2328.61 | 2231.30 |
| Nautilus_IV | 2/23/2018 | 21:02 | -63.366 | -64.954 | 6 | 1.241 | 33.811 | 2340.12 | 2202.57 |
| Nautilus_IV | 2/23/2018 | 23:41 | -63.195 | -64.840 | 5 | 1.074 | 33.744 | 2475.94 | 2231.24 |
| Nautilus_IV | 2/24/2018 | 3:52 | -63.004 | -64.669 | 10 | 0.903 | 33.956 | 2338.07 | 2220.72 |
| Nautilus_V | 1/23/2019 | 19:13 | -63.354 | -64.955 | 6 | 0.641 | 33.429 | 2287.25 | 2215.67 |
| Nautilus_V | 1/23/2019 | 22:04 | -63.203 | -64.836 | 6 | 0.739 | 33.846 | 2337.87 | 2241.51 |
| Nautilus_V | 1/24/2019 | 0:25 | -63.031 | -64.678 | 6 | -0.019 | -0.734 | 2347.31 | 2247.89 |
| Nautilus_V | 1/24/2019 | 5:57 | -62.999 | -64.383 | 5 | 2.317 | 33.710 | 2337.32 | 2182.44 |
| Nautilus_V | 1/24/2019 | 7:24 | -62.909 | -64.467 | 5 | 2.327 | 33.676 | 2294.81 | 2215.73 |
| Nautilus_V | 1/24/2019 | 9:03 | -62.760 | -64.522 | 7 | 1.752 | 33.929 | 2313.25 | 2294.39 |
| Nautilus_V | 1/24/2019 | 10:56 | -62.502 | -64.565 | 6 | 1.247 | 34.053 | 2297.68 | 2342.00 |
| Nautilus_V | 1/24/2019 | 14:42 | -62.108 | -64.420 | 5 | 1.451 | 33.970 | 2343.00 | 2322.00 |
| Nautilus_V | 1/24/2019 | 17:30 | -61.924 | -64.303 | 6 | 1.454 | 33.926 | 2337.49 | 2304.92 |
| Nautilus_V | 1/24/2019 | 19:32 | -61.815 | -64.334 | 6 | 1.688 | 33.880 | 2339.81 | 2281.53 |
| Nautilus_V | 1/24/2019 | 21:11 | -61.741 | -64.373 | 6 | 2.144 | 33.406 | 2318.46 | 2140.64 |
| Nautilus_V | 1/25/2019 | 5:58 | -61.584 | -64.226 | 6 | 1.706 | 33.902 | 2293.22 | 2255.86 |
| Nautilus_V | 1/25/2019 | 7:57 | -61.397 | -64.165 | 6 | 1.721 | 33.787 | 1990.97 | 2008.46 |
| Nautilus_V | 1/25/2019 | 12:39 | -60.783 | -63.951 | 6 | 0.066 | 34.350 | 2357.17 | 2301.77 |
| Nautilus_V | 1/25/2019 | 18:46 | -61.397 | -63.719 | 6 | 1.340 | 34.004 | 2329.49 | 2235.47 |
| Nautilus_V | 1/27/2019 | 15:44 | -64.425 | -64.934 | 5 | 1.742 | 33.629 | 2413.00 | 2152.72 |
| Nautilus_V | 1/27/2019 | 18:52 | -64.015 | -64.840 | 6 | 0.823 | 33.736 | 2417.71 | 2228.16 |
| Nautilus_V | 1/27/2019 | 20:32 | -63.742 | -64.890 | 5 | 0.828 | 33.988 | 2418.81 | 2223.40 |
| Nautilus_V | 1/29/2019 | 22:40 | -62.115 | -64.425 | 5 | 1.701 | 33.915 | 2310.61 | 2157.21 |
| Nautilus_V | 1/30/2019 | 0:51 | -61.832 | -64.335 | 6 | 2.067 | 33.848 | 2327.92 | 2294.69 |
| Nautilus_V | 1/30/2019 | 2:48 | -61.595 | -64.227 | 6 | 1.724 | 33.777 | 2313.27 | 2238.48 |
| Nautilus_V | 1/30/2019 | 4:47 | -61.398 | -64.153 | 6 | 1.364 | 33.814 | 2319.55 | 2229.01 |

**Supplementary References**

1. Rysgaard. S. et al. Sea ice contribution to the air–sea CO_2_ exchange in the Arctic and Southern Oceans. Tellus. 63B, 823–830 (2011).

2. Zeebe, R. E. History of seawater carbonate Chemistry, atmospheric CO_2_ and ocean acidification. Annu. Rev. Earth Planet. Sci. 40, 141–165 (2012).

3. Wanninkhof, R. et al. Ocean acidification along the Gulf Coast and East Coast of the USA. Cont. Shelf Res. 98, 54–71 (2015).

4. Friis, K., A. Körtzinger, and D. W. R. Wallace. The salinity normalization of marine inorganic carbon chemistry data. Geophys. Res. Lett., 30(2), 1085 (2003).

5. Rivaro, P. et al. Distribution of dissolved labile and particulate iron and copper in Terra Nova Bay polynya (Ross Sea, Antarctica) surface waters in relation to nutrients and phytoplankton growth. Continental Shelf Research. 31, 879–889 (2011).

6. Rivaro, P. et al. Distribution of total alkalinity and pH in the Ross Sea (Antarctica) waters during austral summer 2008. Polar Research, 33, 20403 (2014).

7. C.R.B. Mendes, et al. Impact of sea ice on the structure of phytoplankton communities in the northern Antarctic Peninsula. Deep Res. Part II Top. Stud. Oceanogr., 149, 111–123 (2018).

8. Ackley, S. F., Buck K. R. and Taguchi, S. Standing crop of algae in the sea ice of Weddell Sea region. Deep-Sea Research Part I. 26, 269–281 (1979).

9. Monteiro, T., Kerr, R., Orselli, I. B. M., and Lencina-Avila, J. M. Towards an intensified summer CO_2_ sink behaviour in the Southern Ocean coastal regions. Prog. Oceanogr. 183, 102267 (2020).

10. Meredith, M. P. et al. Variability in the freshwater balance of northern Marguerite Bay, Antarctic Peninsula: Results from δ18O. Deep Res. Part II Top. Stud. Oceanogr. 55, 309–322 (2008).

11. Schlitzer, R. Ocean Data View, v. 4.6.3, http://odv.awi.de (2014)

12. Reynolds, R. W. et al. Daily high‐resolution‐blended analyses for sea surface temperature. Journal of Climate. 20(22), 5473–5496 (2007).
